# Extracting stability increases the SNP heritability of emotional problems in young people

**DOI:** 10.1038/s41398-018-0269-5

**Published:** 2018-10-17

**Authors:** Rosa Cheesman, Major Depressive Disorder Working Group of the Psychiatric Genomics Consortium, Kirstin L. Purves, Jean-Baptiste Pingault, Gerome Breen, Fruhling Rijsdij k, Robert Plomin, Thalia C. Eley

**Affiliations:** 1 Social, Genetic and Developmental Psychiatry Cent re, Institute of Psychiatry, Psychology and Neuroscience, King’s College London, London, UK; 2 Division of Psychology and Language Sciences, University College London, London, UK; 3 NIHR Biomedical Research Centre for Mental Health; South London and Maudsley NHS Trust, London, SE5 8AF UK

## Abstract

Twin studies have shown that emotional problems (anxiety and depression) in childhood and adolescence are moderately heritable (~20–50%). In contrast, DNA-based ‘SNP heritability’ estimates are generally <15% and non-significant. One notable feature of emotional problems is that they can be somewhat transient, but the moderate stability seen across time and across raters is predominantly influenced by stable genetic influences. This suggests that by capturing what is in common across time and across raters, we might be more likely to tap into any underlying genetic vulnerability. We therefore hypothesised that a phenotype capturing the pervasive stability of emotional problems would show higher heritability. We fitted single-factor latent trait models using 12 emotional problems measures across ages 7, 12 and 16, rated by parents, teachers and children themselves in the Twins Early Development Study sample. Twin and SNP heritability estimates for stable emotional problems (*N* = 6110 pairs and 6110 unrelated individuals, respectively) were compared to those for individual measures. Twin heritability increased from 45% on average for individual measures to 76% (se = 0.023) by focusing on stable trait variance. SNP heritability rose from 5% on average (n.s.) to 14% (se = 0.049; *p* = 0.002). Heritability was also higher for stable within-rater composites. Polygenic scores for both adult anxiety and depression significantly explained variance in stable emotional problems (0.4%; *p* = 0.0001). The variance explained was more than in most individual measures. Stable emotional problems also showed significant genetic correlation with adult depression and anxiety (average = 52%). These results demonstrate the value of examining stable emotional problems in gene-finding and prediction studies.

## Introduction

Anxiety and depression, known collectively as emotional problems, are highly prevalent in childhood and adolescence (median prevalence 2–24% for anxiety and 0.2–17% for depression)^1^. The average age of onset for anxiety is strikingly young (between 11 and 14), and 50% of anxiety disorders begin before age 11^2^. Similarly, 25% of depression cases are diagnosed before age 19^2^. Emotional problems often take a chronic course throughout life^3^, and predict numerous difficulties in adulthood: anxiety^4^, bipolar disorder^5^, disruptive disorders and schizophrenia^2^. Given that emotional problems are common and disabling, it is essential to unravel their origins in childhood to identify risk factors and develop preventative methods and treatments.

Twin studies have shown that emotional problems in childhood and adolescence are moderately heritable (~20–50%)^6–10^. In contrast, DNA-based ‘SNP heritability’ estimates for childhood emotional problems are generally low and non-significant (in an underpowered and inconsistent literature—see Supplementary Table 1)^11^. Low SNP heritability estimates suggest that genome-wide association studies will struggle to shed light on the genetic architecture of early emotional problems, because both SNP heritability and genome-wide association studies are limited to the additive effects of measured common variants (single-nucleotide polymorphisms (SNPs)). Association studies seeking to identify genetic variants influencing the heritability of childhood anxiety and depression have so far been underpowered and unsuccessful^12–14^. Also, polygenic scores (which aggregate the effects of thousands of genetic variants into a single index of risk for each individual) for a range of traits explain <1% of the variance in emotional problems in childhood and adolescence^15–17^.

A potential way to improve power to identify significant SNP heritability is to tap into the genetic core of emotional problems by assessing problems that are stable across time. Longitudinal twin studies have shown that emotional problems are only moderately stable across childhood and adolescence, but that stability is predominantly influenced by stable genetic influences^18–23^. Moreover, longitudinally assessed stable anxiety traits are more heritable than anxiety at a single time-point^24–26^. In one study, the heritability of adolescents’ stable trait anxiety sensitivity across ages 14, 15 and 17 was 61%, whereas age-specific heritability was zero and non-significant apart from at one age^24^.

Aggregation across reporters and across measures also yields a more heritable core phenotype. Twin research suggests that variation in the part of children’s behaviour that raters see in common captures more of the genetic action than rater-specific variation^27^. For example, two studies found higher twin heritability for rater-common than rater-specific parts of variance in childhood anxiety (~46% vs ~17%)^28,29^. Further, the substantial covariation between anxiety and depression in childhood and adolescence is largely genetically influenced^10,30^.

Analysing phenotypic measurements can also increase heritability through improved reliability. Latent trait modelling allows us to account for measurement error, and more robustly summarise measurements of the same latent characteristics across time, measures and raters on one scale. In genetic research, reducing measurement error variance by definition reduces environmental variance. This increases the proportion of phenotypic variance explained by genetic variance—heritability (both twin and SNP). Previous studies have used latent trait modelling including factor analysis^31,32^ and Item Response Theory^33^ and obtained more reliable psychopathology measures, from which more accurate heritability estimates can be derived, for example, a latent ‘general childhood psychopathology’ factor had a high SNP heritability of 38%, reflecting pervasiveness across domains and raters, as well as lower error^32^. However, no research thus far has applied latent modelling approaches to longitudinal multi-rater emotional problems data, and estimated both twin and SNP heritability.

Our primary hypothesis was that a phenotype capturing the stability of emotional problems across ages, across measures, and across raters would yield higher heritability than individual anxiety and depression measures. To test our hypothesis, we estimated twin and SNP heritabilities of stable emotional problems phenotypes derived from latent modelling. The use of measures assessing both anxiety and depression across multiple raters increases the likelihood of capturing stability in the emotional problems trait, free of rater- and measure-specific views and bias. By ‘stability’, we therefore refer to trait variance in childhood emotional problems that is shared across time, situations (raters), and measures.

Our secondary hypothesis was that the heritability of a stable factor based on all questionnaire items would yield higher heritability than a scale-level approach. Items have unique properties, so heritability can be assessed more accurately by optimally weighting items and reducing the proportion of variance accounted for by item-specific measurement error^33^. To test this, we compared the twin and SNP heritabilities of factor scores derived from two complementary methods: scale-level Confirmatory Factor Analysis (CFA) and Item Response Theory (IRT). In supplementary analyses, we assessed whether heritability was also higher for a crude composite constructed without latent modelling. We compared the independent contributions of stability across age and across raters by analysing cross-age composites for each rater and cross-rater composites for each age. We also investigated the extent that the heritability of stable emotional problems is inflated by individuals with persistent, severe symptoms.

In sum, the present study estimates twin and SNP heritability of a stable emotional problems phenotype constructed from 12 measures from three ages and three raters. These are compared to the twin and SNP heritabilities of the individual anxiety and depression measures. We also extend previous work by assessing the prediction of our stable emotional problems phenotype by polygenic scores for adult anxiety (UK Biobank)^34^ and major depression (PGC)^35^. Additionally, we report results from genome-wide genetic correlation analyses. Our research therefore sheds light on both the genetic architecture of early emotional problems, and links with adult emotional problems.

## Materials and methods

### Sample

The sample is from the Twins Early Development Study (TEDS), a multivariate, longitudinal study of >10,000 twin pairs representative of England and Wales, recruited 1994–1996^36^ Analyses were conducted on a sub-sample of unrelated individuals with available emotional problem data and genome-wide genotyping, plus their co-twins (6110 pairs). Informed consent was obtained from all subjects.

### Genotyping

Full details are in the Supplementary Information. Genotypes were obtained using the AffymetrixGeneChip 6.0 (*N* = 3665) and HumanOmniExpressExome-8v1.2 arrays (*N* = 4649). Typical quality control procedures were followed (e.g., samples were removed based on call rate <0.99, SNPs were removed if minor allele frequency was <0.5%). Genotypes from the two platforms were separately imputed and then merged.

### Measures

**Fig. 1:**
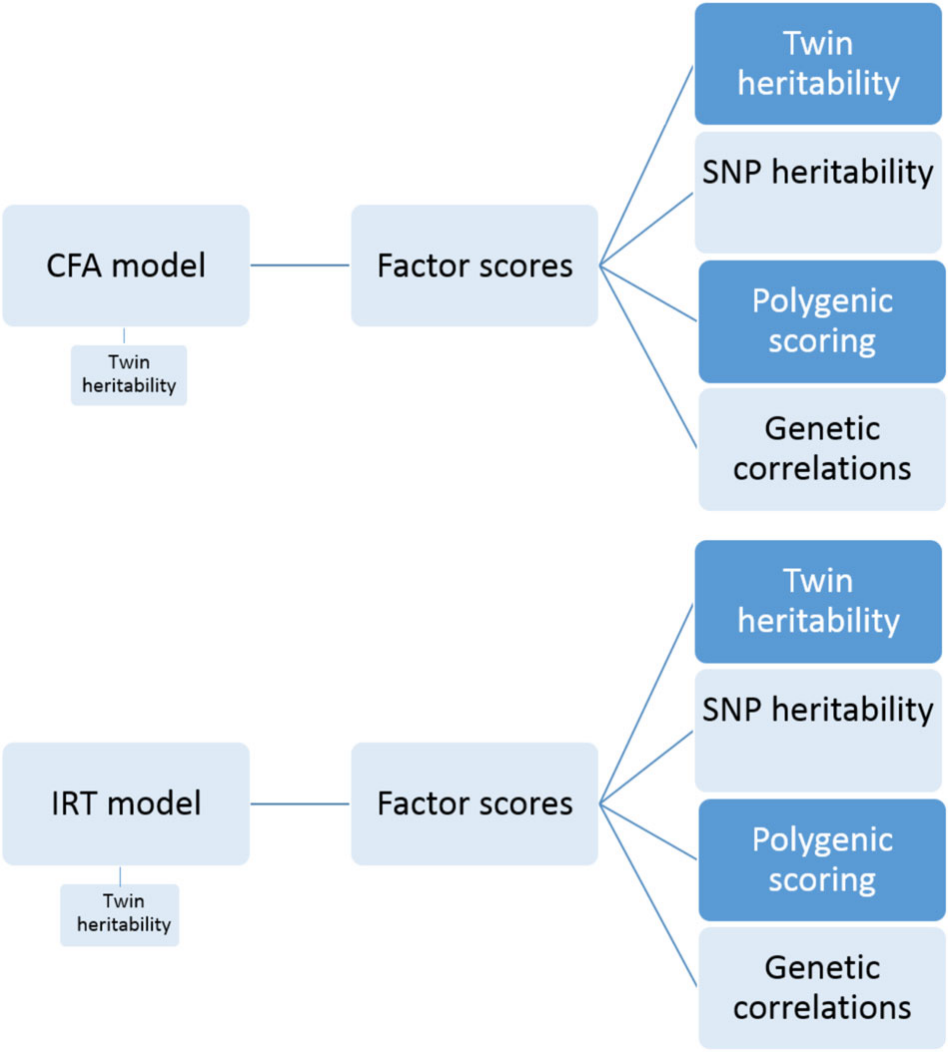
**Workflow diagram of current analyses**

All anxiety and depression variables from ages 7, 12 and 16, and from self-, parent- and teacher ratings, were included. Across these ages, anxiety and depression were measured with 12 scales. See Supplementary Table 2 and Fig. 1 for descriptive statistics and histograms of the scales.

The emotional problems subscale of the Strengths and Difficulties Questionnaire (SDQ)^37^ at ages 7, 12 and 16 was completed by parents^7,12^, teachers^7,12^ and children themselves^12,16^.

The Moods and Feelings Questionnaire (MFQ)^38^ (self- and parent-rated) assessed depressive symptoms at ages 12 and 16. Note that for all MFQ measures except self-rated at age 16, 11 rather than 13 items were collected, due to similarity to SDQ emotional problems items.

The Anxiety-Related Behaviours Questionnaire (ARBQ)^6^ was administered at age 16 to assess parent ratings of anxiety symptoms, behaviour, emotions and cognition.

The Childhood Anxiety Sensitivity Index (CASI)^39^ was used at age 16 to measure self-rated anxiety sensitivity (i.e. fear of the experience of anxiety, and the belief that anxiety has negative consequences).

### Overall scale scores

Total scores were derived for each scale by taking a mean of the items, requiring at least half the items to be present (e.g. at least 9 for the 18-item CASI). All items had three response categories, taking integer values 0–2. Hence each scale has a range of values from 0 to (2 × number of items).

### Statistical analyses

#### Overview

Figure 1 depicts our workflow. We used two latent trait approaches to model our 12 longitudinal multi-rater measures: CFA at the scale level, and IRT at the item-level. In the first stage, we fitted latent trait models to reflect a stable emotional problems factor, accounting for the structure of the twin data (allowing twin heritability to be estimated). In stage two, we extracted individual factor scores for both twins in pair from this latent model (*N* = 6110). Stage three involved four analyses using the stable emotional problems factor scores: (i) estimating twin heritability; (ii) estimating SNP heritability; (iii) predicting stable emotional problems with polygenic scores for adult emotional problems; and (iv) genome-wide association analysis of stable emotional problems. In the fourth stage, we used summary statistics from GWA to explore genetic correlations between stable emotional problems and adult psychopathology. Each stage is explained further below.

To test whether heritability results held with a simpler, non-latent modelling approach, and without combining raters, we conducted sensitivity analyses using simple mean composites across all 12 variables, across age for each variable, and across variables at each age (see Supplementary Figure 4). We also tested whether heritability results held when individuals with persistent severe problems were excluded (see Supplementary Information).

#### Confirmatory Factor Analysis (CFA)

**Fig. 2:**
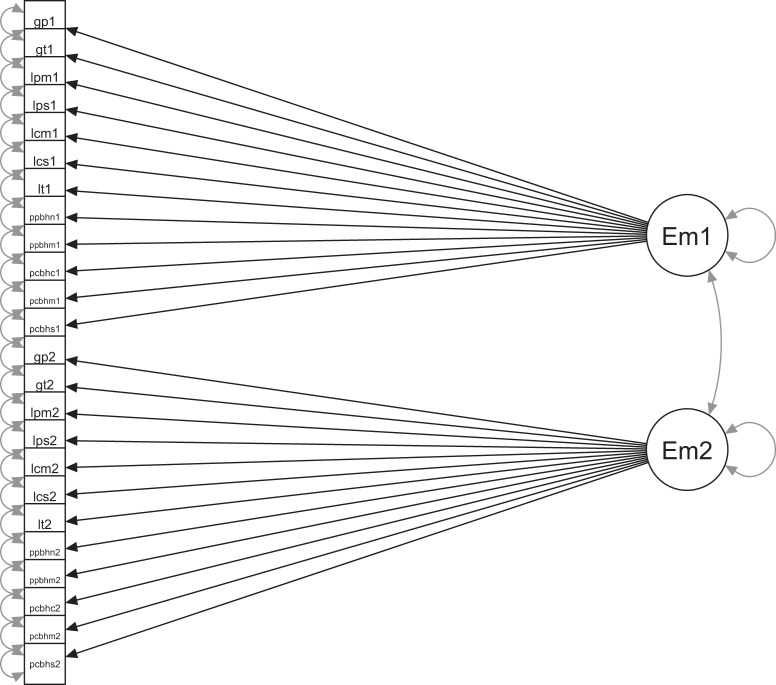
A simplified diagram of our CFA model. Note: Em1 and Em2 are latent emotional problem factors for twin 1 and twin 2. The 24 left-hand variables are observed factor indicators (for each twin), with their variable names as labels (e.g. gp1 = parent-rated SDQ at 7 for twin 1, gp2 = parent-rated SDQ at 7 for twin 2). Twin pairs were allowed to correlate at the latent factor level (shown) and at the scale level (not shown in this figure). Both factors were constrained to a standard normal distribution of mean zero and variance one. Consequently, thresholds, factor loadings and residual variances were all freely estimated, but equated across twins

Figure 2 presents our twelve-indicator one-factor CFA model for twin pairs. The model contains 12 observed continuous variables for each twin of a pair. We included all 12 measures across ages and raters. Two correlated latent emotional problems factors are each measured by the 12 observed factor indicators.

The model was estimated in Mplus, taking into account within-twin-pair correlations at the factor level and at the scale level (*N* = 6110 pairs). Individuals’ factor scores were extracted for estimation of twin and SNP heritability.

The CFA model with twin data also allowed us to obtain twin heritability estimates directly—i.e. by simultaneously estimating the latent trait model and directly decomposing the variance of two latent factors (one for each twin) into genetic and environmental components. In Supplementary Table 7 we present the estimates from simultaneous latent trait twin analyses, plus twin heritability estimates for extracted factor scores. The latter approach can introduce more error than simultaneous modelling^33^.

#### Item Response Theory (IRT)

IRT models describe the relationship between individuals’ responses to specific questionnaire items and their level of the ‘latent variable’ being measured. In the standard two-parameter IRT model, the parameters (item difficulty and discrimination) are equivalent to thresholds and factor loadings in CFA. However, IRT involves categorical observed variables and logistic regressions (rather than continuous and linear). We used all 112 items from the 12 longitudinal cross-rater measures of anxiety and depression in an IRT model. A diagram would look the same as the CFA model in Fig. 1, but with 112 rather than 12 factor indicators for each twin of a pair. Sample size was the same as for CFA.

#### Heritability analyses of factor scores

Factor scores obtained from the above models for 6110 twin pairs were used to estimate twin and SNP heritabilities. For comparison, we estimated twin and SNP heritabilities for the 12 individual measures.

In the twin design, differences in within-pair correlations for MZ and DZ twins are used to estimate genetic, shared environmental and non-shared environmental effects on traits. Greater MZ than DZ similarity indicates genetic influence. Within-pair similarity that is not due to genetic factors is attributed to shared environmental influences. Non-shared environment accounts for individual-specific factors that influence differences among siblings from the same family, plus measurement error. Twin model fitting analysis using full-information maximum likelihood was carried out with structural equation modelling software OpenMx in R^40^.

SNP heritability analyses were conducted using one twin of each pair. Analyses were restricted to individuals with available genotyping and factor scores. During estimation of SNP heritability, individuals with missing covariates and excessive genetic relatedness were removed (one from each pair of individuals with pairwise identity-by-descent (IBD) of >0.025 (third degree relatives)), reducing the final samples to 6002 and 6001, respectively.

SNP heritability was estimated using genomic relatedness matrix restricted maximum likelihood (GREML), implemented in the Genome-wide Complex Trait Analysis (GCTA) program^41^. For this method, we calculate genetic similarity for each pair of unrelated individuals across all genotyped SNPs. To decompose trait variance, genetic similarity is used to predict phenotypic similarity. GCTA only detects additive genetic effects tagged by common SNPs (here, allele frequencies >5%) in our DNA arrays; the residual component includes any other source of variance, including non-additive genetic effects, rare variants, environment, gene–environment interaction and error. We used sex and the first 10 principal components as covariates. See Supplementary Information for details.

#### Polygenic score analyses

Polygenic scores aggregate the effects of thousands of SNPs from genome-wide association studies, including variants that do not achieve genome-wide significance, to provide individual-specific ‘genetic propensity’ estimates. An individual’s polygenic score is the sum of their allele count weighted by the effect size for each SNP, as derived from GWAS. We used the high-resolution approach in PRSice 2^42^ to obtain the most predictive polygenic score (with the best *p*-value threshold for inclusion of SNPs) for each phenotype. Our analyses included 10,000 permutations to obtain more stringent empirical *p*-values.

In this study, we generated polygenic scores using summary statistics from PGC Depression (130,664 MDD cases and 330,470 controls; without 23&Me—results based on summary statistics for a subset of 10,000 variants in a sample including 23&Me are reported in Supplementary Table 8)^35^ and UK Biobank case–control anxiety (25,453 probable Generalised Anxiety Disorder cases and 58,113 controls)^34^ genome-wide association studies. We predicted phenotypic variance in the CFA- and IRT-derived stable emotional problems scores with the polygenic scores for depression and anxiety. To compare the prediction of the stable scores with that of individual measures, the 12 individual measures were also regressed on the two polygenic scores. We used sex and the first 10 principal components as covariates.

#### Genome-wide association analysis

Genome-wide association analysis was performed on the CFA-derived stable emotional problems phenotype (*N* = 6110 individuals) using PLINK v1.90b3.31^43^.

Subsequent analyses were conducted using the summary statistics: calculation of genetic correlations with UK Biobank anxiety and PGC depression using LD Score Regression 44 (color and linked) and with a range of phenotypes from publicly available external GWAS using LD Hub^45^.

### Code availability

Computer code used in our analyses is available from the authors upon request.

## Results

### Latent trait analyses

A CFA single-factor model using 12 longitudinal multi-rater emotional problems measures was fitted to the data. All scales loaded significantly on the stable emotional problems factor (*p* < 0.0005). As expected given the low stability of anxiety and depression across childhood (see Supplementary Figure 2 for phenotypic correlations), model fit was relatively poor. However, our aim was to extract a stable developmental factor and estimate its heritability, and not to offer the best explanation of covariance. See Supplementary Tables 4–6 for CFA results.

### Heritability analyses

**Fig. 3:**
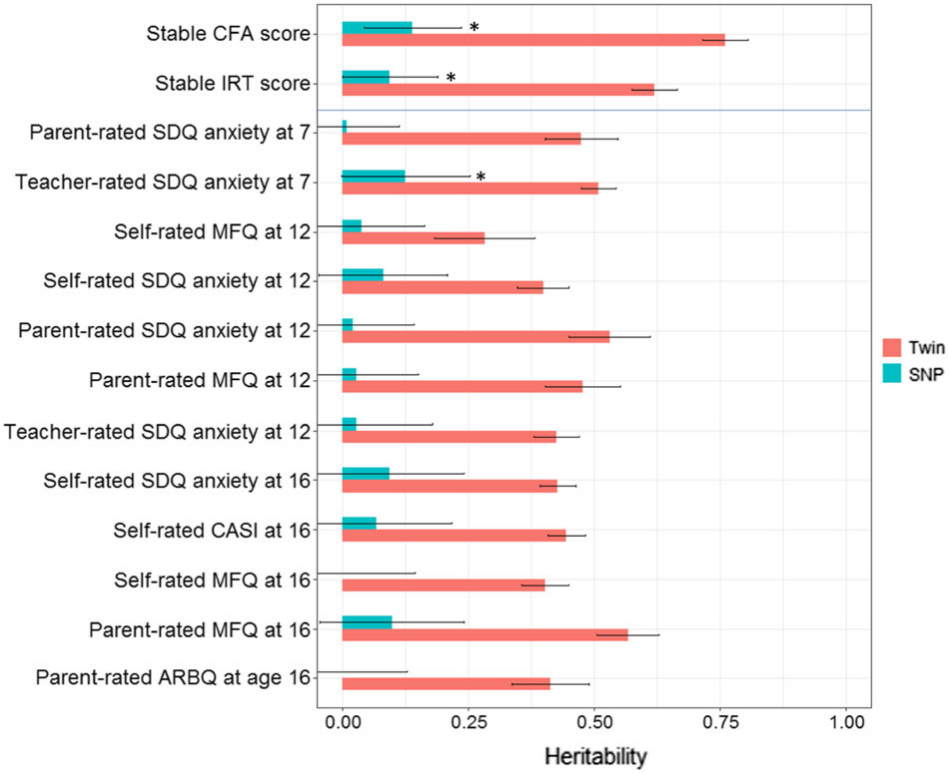
Twin and SNP heritabilities of the CFA- and IRT-derived scores for stable emotional problems, and of the 12 individual measures (all with 95% confidence intervals). Note: sample sizes were 6110 for twin analyses; 6002 and 6001 for SNP heritability analyses of CFA and IRT stable emotional problems scores, respectively. ‘*’ indicates statistically significant SNP heritability estimates (*p* < 0.05)

Results support our hypothesis that extracting stable variance increases the heritability of childhood emotional problems. Figure 3 shows that twin heritability increased from 45% on average (range: 28% (se = 0.05; 95% CI = 0.18–0.38) – 57% (se = 0.02; 95% CI = 0.51–0.63)) to 76% (se = 0.02; 95% CI = 0.72–0.81). SNP heritability rose from 5% on average (range: 0% (se = 0.07; 95% CI = −0.15 to 0.15) –13% (se = 0.07; 95% CI = −0.001 to 0.25)) to 14% (se = 0.05; 95% CI = 0.04–0.24; *p* = 0.002). For most individual anxiety and depression measures, point estimates are low and the intervals based on standard errors cross zero. None of these individual measures had a significant SNP heritability (*p* < 0.05), except teacher-rated SDQ at age 7.

Scale- and item-level approaches increased SNP heritability equally. Promisingly, IRT factor scores are more normally distributed (see Supplementary Figure 3), but CFA is easier to understand and execute. We thus used CFA but not IRT factor scores in subsequent analyses. Twin heritability estimates for simultaneous latent trait-twin modelling and extracted factor scores were equivalent: point estimates were almost identical and SE intervals were overlapping. See Supplementary Table 7 for full twin and SNP heritability results. Supplementary Figure 4 contains results from sensitivity analyses confirming that aggregation across age and raters increases heritability even when not using latent modelling to account for unreliability. These results also suggest that heritability is still higher when aggregating across age for each rater, and across raters for each age. We show in Supplementary Information that there is no statistically significant difference in heritability upon removal of individuals with persistently severe emotional problems, although point estimates were lower, so replication of this analysis in an independent sample is needed.

### Polygenic scoring

Polygenic scores for anxiety and depression significantly explain variance in stable emotional problems (Table 1). More variance is explained than in most of the individual measures (~0.4% vs. ~0.2% on average, although comparison of *R*^2^ statistics is difficult, given the differing target sample sizes and the lack of error estimates).

### Genome-wide association

No genome-wide significant SNP associations were identified. See Supplementary Information for Manhattan plots (Supplementary figures 7, 8).

**Table 1:** Proportion of phenotypic variance predicted in stable emotional problems, and in 12 anxiety and depression measures, by polygenic scores for Major Depressive Disorder and for General Anxiety Disorder

	*R* ^2^		*p*-value		Empirical *p*-value	
	PGC MDD	UKBB ANX	PGC MDD	UKBB ANX	PGC MDD	UKBB ANX
Stable CFA score	0.0048	0.0041	<0.0001	<0.0001	0.0001*	0.0001*
Stable IRT score	0.0042	0.0035	<0.0001	<0.0001	0.0001*	0.0001*
Parent-rated SDQ at 7	0.0007	0.0012	0.0481	0.0115	0.1305	0.038*
Teacher-rated SDQ at 7	0.0031	0.003	0.0002	0.0003	0.0013*	0.0010*
Self-rated MFQ at 12	0.0041	0.0008	<0.0001	0.0544	0.0002*	0.1623
Self-rated SDQ at 12	0.001	0.0022	0.0362	0.0014	0.1041	0.0046*
Parent-rated SDQ at 12	0.0017	0.0015	0.0054	0.0087	0.0186*	0.0313*
Parent-rated MFQ at 12	0.0015	0.0025	0.0086	0.0021	0.0277*	0.0030*
Teacher-rated SDQ at 12	0.0008	0.0012	0.0791	0.0019	0.2087	0.034*
Self-rated SDQ at 16	0.0015	0.0031	0.0106	0.0002	0.0325*	0.0005*
Self-rated CASI at 16	0.003	0.0024	0.0006	0.0013	0.0022*	0.0054*
Self-rated MFQ at 16	0.0018	0.0013	0.0075	0.0241	0.0217*	0.0771*
Parent-rated MFQ at 16	0.003	0.0016	0.0007	0.0135	0.0023*	0.0135*
Parent-rated ARBQ at 16	0.003	0.0017	0.0004	0.0089	0.0017*	0.0300*

Note: *R*^2^ indicates proportion of variance predicted by each of the two polygenic scores in the 2 stable and 12 individual phenotypes; *PGC MDD* polygenic scores for Depression (from the PGC without 23&Me data); *UKBB ANX* polygenic scores for Anxiety (from UK Biobank); Empirical *p*-values were obtained from 10,000 permutations to correct for multiple testing of *p*-value thresholds, and ‘*’ indicates target TEDS phenotypes that were significantly predicted by polygenic scores at the more stringent empirical *p*-value thresholds; Supplementary Figures 5, 6 show that prediction is reliable across multiple thresholds; Supplementary Table 8 shows that similar results were obtained using summary stats for 10,000 SNPs from a larger sample including 23&Me data

Our stable emotional problems score showed significant genetic correlations with adult depression (case–control (0.48) and symptoms (0.64)), case–control anxiety (0.45), and wellbeing (−0.36). The latter did not remain significant after multiple testing correction, and genetic correlations with nine other phenotypes were non-significant (see Supplementary Table 9).

## Discussion

This study found that a phenotype capturing the pervasive stability of emotional problems across childhood and adolescence showed higher heritability than individual measures. Twin heritability increased from 45% on average for individual measures to 76% (se = 0.02; 95% CI = 0.72–0.81) for stable emotional problems. SNP heritability rose from 5% on average to 14% (se = 0.05; 95% CI = 0.04–0.24; *p* = 0.002) by capturing common variance across ages and raters. These findings were consistent for a common factor based on scale-level and item-level data for the 12 measures. The findings also held for a simple, non-latent variable approach. This simple approach is easier to calculate but does not account for measurement error. Additionally, polygenic scores for adult anxiety and depression significantly explained variance in stable emotional problems, and the variance explained (0.4% (*p* = 0.0001) was higher than in most individual measures. Stable emotional problems showed significant genetic correlation with adult depression and anxiety (average = 52%), mirroring previous findings in adults^34,46^. Together, the polygenic score and genetic correlation results demonstrate that a significant proportion of common SNPs influencing stable emotional problems in childhood also influence adult anxiety and depression.

This research has several limitations. Our hypothesis was concerned with capturing stability in emotional problems, and not with finding the best-fitting model explaining stability and change across age, and the structure of age-, scale- and rater-specific influences. Consequently, we did not explicitly separate out transient and enduring factors, but acknowledge that the presence of genes driving stable emotional problems does not preclude age effects. We also note the contribution of stability in measurement, not only of longitudinal stability, to increased heritability, although heritability remains higher for stable within-rater composites (Supplementary Figure 4). Structural equation models such as the Trait–State–Occasion (TSO) model^47^ can be used to explicitly separate these effects. However, our inclusion of multi-rater data is an advantage of this study. The common variance that has been extracted is free of rater bias or rater-specific views. Our factor scores therefore reflect a core emotional problems trait: longitudinally stable and agreed upon by three raters.

Our findings suggest that common genetic variants have stable influences on emotional problems throughout childhood and adolescence, which extend into later life. Additional research from large longitudinal studies is needed to determine whether the SNP heritability of emotional problems is more accurately estimated by capturing pervasive stability beyond age 16, or even throughout the life course. This is likely, given the evidence from twin research that genetic influences on emotional problems at age 3 remain influential well into adulthood^18^. Further research could also investigate whether extracting a measure of stability increases the heritability of other less developmentally stable and less heritable traits.

Future genomic studies of emotional problems could benefit from adopting a lifelong approach, using measures of adult case/control status as well as childhood dimensions. Our results indicate that stable emotional problems offer a more useful phenotype than individual measures for: finding variants predisposing to early emotional problems; creating polygenic scores for emotional problems in childhood and adolescence; and for using as a target phenotype for polygenic prediction. Subsequent research should examine how polygenic risk for stable child and adolescent emotional problems is expressed throughout development and into adulthood, as well as the mediators, moderators and multivariate outcomes of this polygenic risk.

The young age of onset of emotional problems, and their persistent, wide-ranging negative outcomes, mean that prediction and prevention should be prioritised. The key contribution of genomic research into early emotional problems is likely to be the predictive value of polygenic scores. The predictive accuracy of polygenic scores is increasing with gains in power for genome-wide association, thanks to collaborative consortia and large national projects with homogenous phenotyping such as the UK Biobank. Researchers are working towards the much-needed large-scale genomic study of emotional problems in childhood itself. The present research underlines the utility of extracting a more stable emotional problems phenotype. This phenotype, theoretically grounded in evidence from decades of twin research, is more reliable, heritable and useful for prediction studies.

## Electronic supplementary material

Supplementary Information

## References

[1] Merikangas KR, Nakamura EF, Kessler RC (2009). Epidemiology of mental disorders in children and adolescents. Dialog. Clin. Neurosci..

[2] Kessler RC (2005). Lifetime prevalence and age-of-onset distributions of DSM-IV disorders in the National Comorbidity Survey Replication. Arch. Gen. Psychiatry.

[3] Beesdo-Baum K, Knappe S (2012). Developmental epidemiology of anxiety disorders. Child Adolesc. Psychiatr. Clin. N. Am..

[4] Copeland WE, Angold A, Shanahan L, Costello EJ (2014). Longitudinal patterns of anxiety from childhood to adulthood: the Great Smoky Mountains Study. J. Am. Acad. Child Adolesc. Psychiatry.

[5] Cohen D (2000). Absence of cognitive impairment at long-term follow-up in adolescents treated with ECT for severe mood disorder. Am. J. Psychiatry.

[6] Eley TC (2003). A twin study of anxiety-related behaviours in pre-school children. J. Child Psychol. Psychiatry.

[7] Saudino KJ, Carter AS, Purper-Ouakil D, Gorwood P (2008). The etiology of behavioral problems and competencies in very young twins. J. Abnorm. Psychol..

[8] Van Hulle CA, Lemery-Chalfant K, Goldsmith HH (2007). Genetic and environmental influences on socio-emotional behavior in toddlers: an initial twin study of the infant-toddler social and emotional assessment. J. Child Psychol. Psychiatry.

[9] Hallett V, Ronald A, Rijsdijk F, Eley TC (2009). Phenotypic and genetic differentiation of anxiety-related behaviors in middle childhood. Depress Anxiety.

[10] Waszczuk MA, Zavos HMS, Gregory AM, Eley TC (2014). The phenotypic and genetic structure of depression and anxiety disorder symptoms in childhood, adolescence, and young adulthood. JAMA Psychiatry.

[11] Cheesman R (2017). Childhood behaviour problems show the greatest gap between DNA-based and twin heritability. Transl. Psychiatry.

[12] Trzaskowski M (2013). First genome-wide association study on anxiety-related behaviours in childhood. PLoS ONE.

[13] Mick E (2011). Genome-wide association study of the child behavior checklist dysregulation profile. J. Am. Acad. Child Adolesc. Psychiatry.

[14] Benke KS (2014). A genome-wide association meta-analysis of preschool internalizing problems. J. Am. Acad. Child Adolesc. Psychiatry.

[15] Krapohl E (2016). Phenome-wide analysis of genome-wide polygenic scores. Mol. Psychiatry.

[16] Jones HJ (2016). Phenotypic manifestation of genetic risk for schizophrenia during adolescence in the general population. JAMA Psychiatry.

[17] Nivard MG, Gage SH, Hottenga JJ, van Beijsterveldt CEM, Abdellaoui A, Bartels M, Baselmans BML, Ligthart L, Pourcain BS, Boomsma DI, Munafò MR, Middeldorp CM (2017). Genetic overlap between schizophrenia and developmental psychopathology: longitudinal and multivariate polygenic risk prediction of common psychiatric traits during development.. Schizophrenia Bulletin.

[18] Nivard MG (2015). Stability in symptoms of anxiety and depression as a function of genotype and environment: a longitudinal twin study from ages 3 to 63 years. Psychol. Med..

[19] Trzaskowski M, Zavos HMS, Haworth CMA, Plomin R, Eley TC (2012). Stable genetic influence on anxiety-related behaviours across middle childhood. J. Abnorm. Child Psychol..

[20] Waszczuk MA, Zavos HMS, Gregory AM, Eley TC (2016). The stability and change of etiological influences on depression, anxiety symptoms and their co-occurrence across adolescence and young adulthood. Psychol. Med..

[21] Hannigan LJ, Walaker N, Waszczuk MA, McAdams TA, and Eley TC (2017). Aetiological influences on stability and change in emotional and behavioural problems across development: a systematic review.. Psychopathology review.

[22] Kendler KS (2008). A longitudinal twin study of fears from middle childhood to early adulthood: evidence for a developmentally dynamic genome. Arch. Gen. Psychiatry.

[23] Lau JYF, Eley TC (2006). Changes in genetic and environmental influences on depressive symptoms across adolescence and young adulthood. Br. J. Psychiatry.

[24] Zavos HMS, Gregory AM, Eley TC (2012). Longitudinal genetic analysis of anxiety sensitivity. Dev. Psychol..

[25] Lubke GH (2016). A powerful phenotype for gene-finding studies derived from trajectory analyses of symptoms of anxiety and depression between age seven and 18. Am. J. Med. Genet. B. Neuropsychiatr. Genet..

[26] Hatoum AS, Rhee SH, Corley RP, Hewitt JK, Friedman NP (2018). Etiology of stability and growth of internalizing and externalizing behavior problems across childhood and adolescence. Behav. Genet..

[27] Bartels M (2004). Disentangling genetic, environmental, and rater effects on internalizing and externalizing problem behavior in 10-year-old twins. Twin. Res..

[28] Wesseldijk LW (2016). Psychopathology in 7-year-old children: Differences in maternal and paternal ratings and the genetic epidemiology. Am. J. Med. Genet. B Neuropsychiatr. Genet..

[29] Fedko IO (2017). Heritability of behavioral problems in 7-year olds based on shared and unique aspects of parental views. Behav. Genet..

[30] Eley TC, Stevenson J (1999). Using genetic analyses to clarify the distinction between depressive and anxious symptoms in children. J. Abnorm. Child Psychol..

[31] Laurin CA, Hottenga JJ, Willemsen G, Boomsma DI, Lubke GH (2015). Genetic analyses benefit from using less heterogeneous phenotypes: an illustration with the hospital anxiety and depression scale (HADS). Genet. Epidemiol..

[32] Neumann A (2016). Single nucleotide polymorphism heritability of a general psychopathology factor in children. J. Am. Acad. Child Adolesc. Psychiatry.

[33] Van den Berg SM, Glas CAW, Boomsma DI (2007). Variance decomposition using an IRT measurement model. Behav. Genet..

[34] Purves, K. L., et al. The common genetic architecture of anxiety disorders. *BioRxiv*. 2017.

[35] Wray NR, Ripke S, Mattheisen M, Trzaskowski M (2018). Genome-wide association analyses identify 44 risk variants and refine the genetic architecture of major depression.. Nature Genetics..

[36] Haworth CMA, Davis OSP, Plomin R (2013). Twins Early Development Study (TEDS): a genetically sensitive investigation of cognitive and behavioral development from childhood to young adulthood. Twin. Res. Hum. Genet..

[37] Goodman R (1997). The strengths and difficulties questionnaire: a research note. J. Child Psychol. Psychiatry.

[38] Angold A, Costello EJ, Messer SC, Pickles A (1995). The development of a questionnaire for use in epidemiological studies of depression in children and adolescents. Int. J. Methods Psychiatr. Res..

[39] Silverman WK, Fleisig W, Rabian B, Peterson RA (1991). Childhood anxiety sensitivity index. J. Clin. Child Psychol..

[40] Boker S (2011). Openmx: an open source extended structural equation modeling framework. Psychometrika.

[41] Yang J, Lee SH, Goddard ME, Visscher PM (2011). GCTA: a tool for genome-wide complex trait analysis. Am. J. Hum. Genet..

[42] Euesden J, Lewis CM, O’Reilly PF (2015). PRSice: Polygenic Risk Score software. Bioinformatics.

[43] Purcell S (2007). PLINK: a tool set for whole-genome association and population-based linkage analyses. Am. J. Hum. Genet..

[44] Bulik-Sullivan Brendan, Finucane Hilary K, Anttila Verneri, Gusev Alexander, Day Felix R, Loh Po-Ru, Duncan Laramie, Perry John R B, Patterson Nick, Robinson Elise B, Daly Mark J, Price Alkes L, Neale Benjamin M (2015). An atlas of genetic correlations across human diseases and traits. Nature Genetics.

[45] Zheng J (2017). LD Hub: a centralized database and web interface to perform LD score regression that maximizes the potential of summary level GWAS data for SNP heritability and genetic correlation analysis. Bioinformatics.

[46] Brainstorm Consortium, Anttila V, Bulik-Sullivan B, Finucane HK (2015). Analysis of shared heritability in common disorders of the brain.. Science.

[47] Cole DA (2006). Coping with longitudinal data in research on developmental psychopathology. Int. J. Behav. Dev..

