# Supplementary material for: Extracting stability increases the SNP heritability of emotional problems in young people
Source: Transl Psychiatry. 2018 Oct 17;8:223. doi: 10.1038/s41398-018-0269-5 (PMC6193004; doi:10.1038/s41398-018-0269-5)
Supplement: Supplementary file 1 — Supplementary Information [file 41398_2018_269_MOESM1_ESM.pdf]

Table of Contents

Table 1: Literature review: SNP heritability of child and adolescent emotional problems 2

Table 2: Descriptive statistics for the 12 TEDS anxiety and depression scales 3

Figure 1: Histograms of TEDS anxiety and depression scales 4

Figure 2: Phenotypic correlations of the 12 anxiety and depression scales at ages 7, 12 and 16 5

Figure 3: histograms of CFA- (left) and IRT-derived (right) stable emotional problems scores 6

CFA model fit and factor loadings 7

Full genotyping information 9

SNP heritability: supplementary methods 10

Table 7: Full twin and SNP heritability results: 11

Figure 4: Twin and SNP heritability results for a simple mean approach to aggregating emotional problems measures, with sensitivity analyses to investigate rater effects 12

Investigation of the contribution of individuals with persistent emotional problems to the increased heritability of our stable phenotype. 14

Figures 5 and 6: Polygenic score results 15

Table 8: variance explained by polygenic scores for MDD, derived from summary statistics for a subset of 10,000 variants in the PGC sample including 23&Me. 16

Figures 7 and 8: Genome-wide association results 17

Table 9: Genetic correlations between stable emotional problems (from CFA) and psychiatric traits, obtained using LD Score regression. 18

Full authors list: Major Depressive Disorder Working Group of the Psychiatric Genomics Consortium 19

LD Hub acknowledgements 25

Table 1: Literature review: SNP heritability of child and adolescent emotional problems

Reference	Year	Trait	Sample	Measure	Age	Reporter	N	SNP heritability	SE	P value
Benke et al.	2014	Emotional problems	Gen R	CBCL 1.5-5 internalising scale	3	Primary caregiver	2037	0.26	NA	0.07
			NTR	CBCL 2-3 internalising scale	3	Mother	1475	0.18	NA	0.3
			Raine	CBCL 2-3 internalising scale	2	Primary caregiver	1084	0.13	NA	0.33
Pappa et al.	2015	Emotional problems	NTR / Gen R	CBCL internalising	7	Parent	3175	0.12	0.1	
Sallis et al.	2017	Depressive Symptoms	ALSPAC	Short Moods and Feelings Questionnaire (SMFQ)	11	Child	5480	0.07	0.1	
					13	Child	5055	0.17	0.1	
					14	Child	4615	0.02	0.1	
					17	Child	3605	0.08	0.1	
					18	Child	3289	0.03	0.1	
					19	Child	2433	0.05	0.1	
					10	Parent	5571	0.04	0.1	
					12	Parent	5119	0.12	0.1	
					13	Parent	5031	0.07	0.1	
					17	Parent	3990	0.04	0.1	

Note: Gen R= Generation R; NTR= Netherlands Twin Register; ALSPAC= Avon Longitudinal Study of Parents and Children; CBCL = Child Behaviour Checklist.

Three studies in samples other than TEDS have estimated SNP heritabilities for quantitative measures of childhood anxiety and depression, known as emotional problems, or internalising'. The average SNP heritability estimate from these studies is 10%. Although this is twice as high as the average in this study from the TEDS sample (5%), 10% SNP heritability is still well below half of the twin estimates of these phenotypes in the literature (40-60% in TEDS). Larger samples are needed to clarify this inconsistent and underpowered literature. For example, the study of pre-school internalising symptoms (Benke et al., 2014) had maximum 35% power with a sample of 2000 if true SNP heritability was half of the twin estimate they cite (50%), and a minimum of 10% power with a sample of 1000 and heritability of 20%. Moreover, the large ranges within the studies makes the estimates difficult to interpret.

Table 2: Descriptive statistics for the 12 TEDS anxiety and depression scales

	**Measure**	**Sample size (twin pairs)**	**Number of items**	**Mean**	**Standard deviation**
1	Parent-rated SDQ at 7	5367	5	2.13	1.80
2	Teacher-rated SDQ at 7	4419	5	1.30	1.77
3	Parent-rated MFQ at 12	4486	11	1.13	2.24
4	Parent-rated SDQ at 12	4487	5	1.77	1.91
5	Self-rated MFQ at 12	4493	11	2.30	3.28
6	Self-rated SDQ at 12	4475	5	2.17	2.05
7	Teacher-rated SDQ at 12	3713	5	1.16	1.77
8	Parent-rated ARBQ at 16	3845	19	3.43	4.08
9	Parent-rated MFQ at 16	3844	11	0.96	2.28
10	Self-rated CASI at 16	3831	18	7.85	5.86
11	Self-rated MFQ at 16	3832	13	3.59	4.37
12	Self-rated SDQ at 16	3830	5	2.76	2.27

Figure 1: Histograms of TEDS anxiety and depression scales

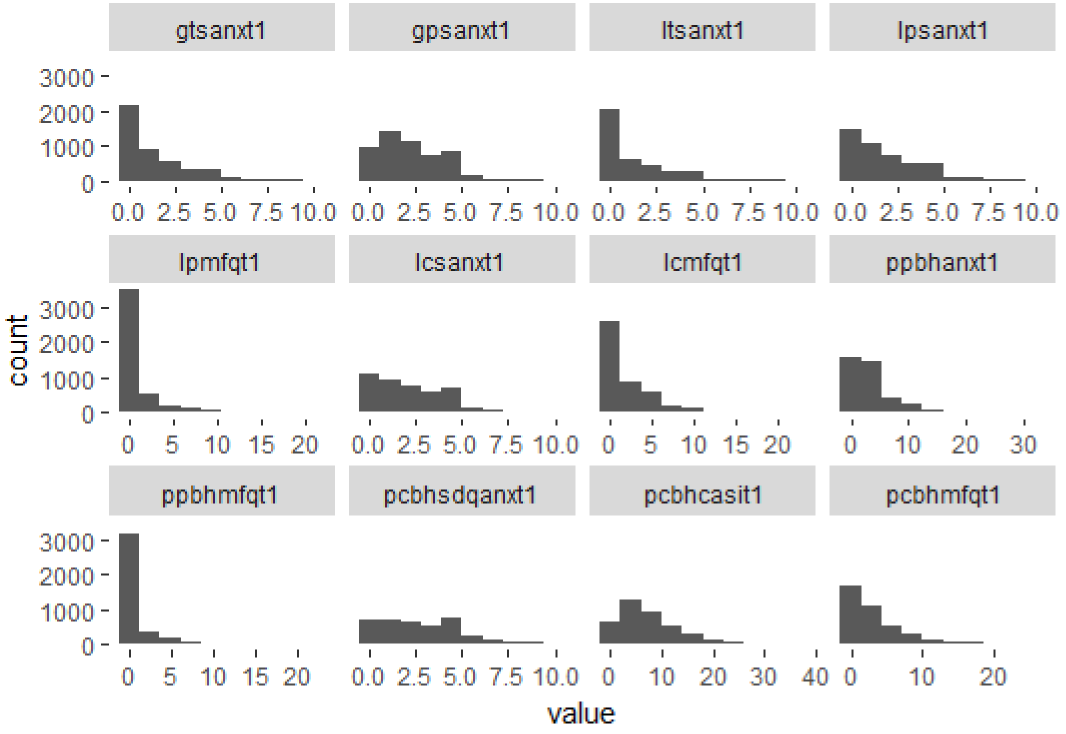

Note: 'gtsanxt1'= Teacher-rated SDQ at 7, 'gpsanxt1'= Parent-rated SDQ at 7, 'ltsanxt1'= Teacher-rated SDQ at 12, 'lpsanxt1' = Parent-rated SDQ at 12, 'lpmfqt1'= Parent-rated MFQ at 12, 'lcsanxt1'= Self-rated SDQ at 12 ,'lcmfqt1'= Self-rated MFQ at 12, 'ppbhanxt1'= Parent-rated ARBQ at 16, 'ppbhmfqt1= Parent-rated MFQ at 16', 'pcbhsdqanxt1'= Self-rated SDQ at 16,'pcbhcasit1'= Self-rated CASI at 16, 'pcbhmfqt1'= Self-rated MFQ at 16.

Figure 2: Phenotypic correlations of the 12 anxiety and depression scales at ages 7, 12 and 16

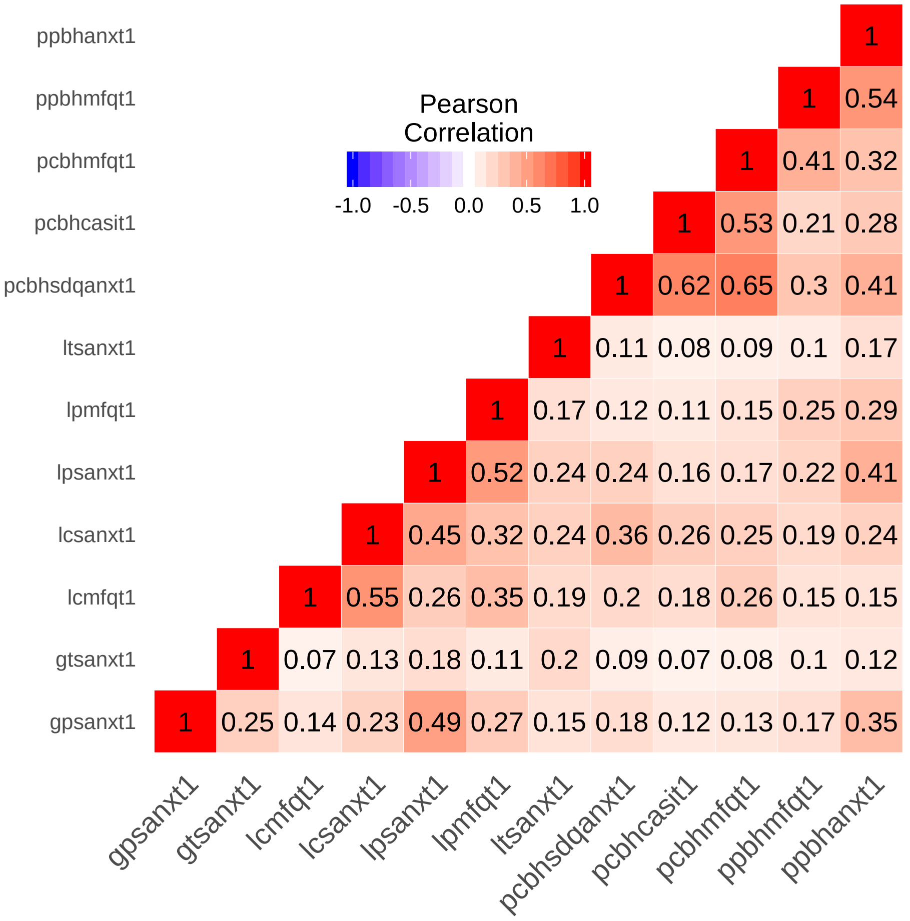

Note: correlations were calculated in R, before any data cleaning, and including only the people with genotype data and complete data for these 12 scales. See Note of Supplementary Figure 1 for variable names.

Figure 3: histograms of CFA- (left) and IRT-derived (right) stable emotional problems scores

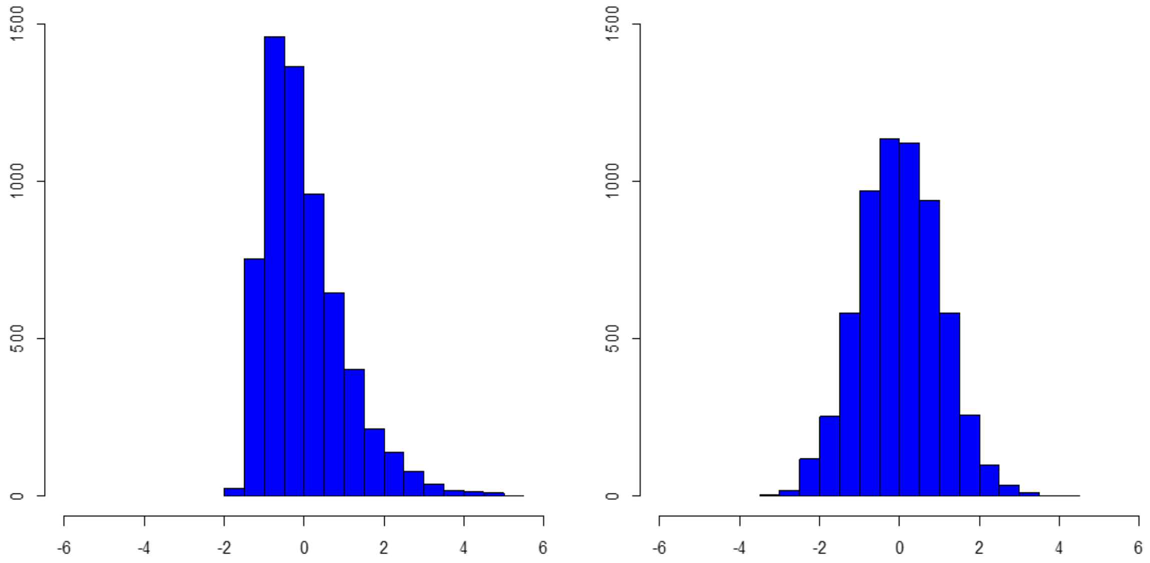

Note that the correlation between CFA and IRT factor scores is 0.92.

CFA model fit and factor loadings

Table 3: Twin correlations for emotional problems stable latent factor from CFA

Twin correlations	
MZ	0.85
DZ	0.489

Table 4: Model Fit

The single-factor model did not provide a good fit to the data. CFI and TLI were less than 0.9, suggesting worse fit than a restricted baseline model, and RMSEA was significantly greater than 0.05, suggesting poor fit. However, poor fit might be expected: it could reflect the lack of stability of anxiety across childhood (as indicated by the low correlations across our measures (heat map above), and the low stability in the literature), or it could reflect the higher than modelled associations within raters and within time-points.

Fit indices for the CFA model of childhood emotional problems	
CFI	0. 68
TLI	0. 69
RMSEA	0.08

Note: RMSEA = Root-Mean-Square Error of Approximation; CFI = Comparative Fit Index; TLI = Tucker-Lewis Index.

Table 5: Standardised Factor Loadings

With regard to the CFA model, all measures load significantly onto the stable emotional problems factor. All measures loaded ≥0.5, apart from the two teacher-rated measures, and self-rated MFQ at 12.

Measure	Factor Loading	SE
Parent-rated SDQ at 7	0.51	0.00
Teacher-rated SDQ at 7	0.42	0.01
Self-rated MFQ at 12	0.33	0.01
Self-rated SDQ at 12	0.61	0.01
Parent-rated SDQ at 12	0.50	0.01
Parent-rated MFQ at 12	0.62	0.01
Teacher-rated SDQ at 12	0.49	0.01
Self-rated SDQ at 16	0.61	0.01

Self-rated CASI at 16	0.56	0.01
Self-rated MFQ at 16	0.73	0.01
Parent-rated MFQ at 16	0.59	0.01
Parent-rated ARBQ at age 16	0.68	0.01

Table 6: Residual variances

Measure	Residual variance	SE
Parent-rated SDQ at 7	0.74	0.00
Teacher-rated SDQ at 7	0.82	0.01
Self-rated MFQ at 12	0.89	0.01
Self-rated SDQ at 12	0.63	0.01
Parent-rated SDQ at 12	0.75	0.01
Parent-rated MFQ at 12	0.61	0.01
Teacher-rated SDQ at 12	0.76	0.01
Self-rated SDQ at 16	0.63	0.01
Self-rated CASI at 16	0.69	0.01
Self-rated MFQ at 16	0.47	0.01
Parent-rated MFQ at 16	0.65	0.01
Parent-rated ARBQ at age 16	0.54	0.01

Full genotyping information

DNA from 3665 individuals extracted from buccal cheek swabs was hybridized to AffymetrixGeneChip 6.0 SNP genotyping arrays using standard WTCCC2 experimental protocols. 4649 other individuals of European ancestry were genotyped on HumanOmniExpressExome-8v1.2 arrays at the Molecular Genetics Laboratories of the Medical Research Council Social, Genetic Developmental Psychiatry Centre, based on DNA extracted from saliva samples.

After initial quality control and genotype calling, the same quality control was conducted on the samples genotyped on the Affymetrix and Illumina platforms separately using PLINK (Purcell et al. 2007; Chang et al. 2015), R (GBIF.ORG n.d.), and vcftools (Danecek et al. 2011). Samples were removed based on call rate (<0.99), suspected non-European ancestry, heterozygosity, array signal intensity, and relatedness. SNPs were excluded if minor allele frequency was <0.5% , if more than 1% of genotype data were missing, or if the Hardy Weinberg p-value was lower than 10-5. Non-autosomal markers and indels were removed. Associations between the SNP and the platform, batch, or plate on which samples were genotyped were calculated; SNPs with p-values less than 10-3 were excluded. A total sample of 6710 individuals remained after quality control – 3093 individuals and 525 859 SNPs genotyped on the Affymetrix platform and 3617 individuals and 600 034 SNPs genotyped on the Illumina platform.

Genotypes from the two platforms were separately imputed using the Haplotype Reference Consortium 5 and Minimac3 1.0.13 (Howie et al. 2012; Fuchsberger et al. 2015) before merging genotype data obtained from both arrays.

We performed principal component analysis on a subset of 42 859 common (MAF>5%) autosomal HapMap3 SNPs (International HapMap 3 Consortium et al. 2010), after stringent pruning to remove markers in linkage disequilibrium (r2 >0.1) and excluding high linkage disequilibrium genomic regions so as to ensure that only genome-wide effects were detected.

SNP heritability: supplementary methods

SNP heritabilities were estimated using genomic relatedness matrix restricted maximum likelihood (GREML), implemented in the Genome-wide Complex Trait Analysis (GCTA) program (Yang et al. 2011). This estimates genetic influence directly using genome-wide genotypes in large samples of unrelated individuals. First, genetic similarity for each pair of unrelated individuals across all genotyped or imputed genetic markers is calculated. Each pair's genetic similarity is then used to predict their phenotypic similarity. In the present study, one from each pair of individuals with pairwise identity-by-descent (IBD) of >0.025 (third degree relatives) were removed, so that chance genetic similarity could be used as a random effect in a linear mixed model. Comparing a matrix of pairwise genomic similarity to a matrix of pairwise phenotypic similarity using a random-effects mixed linear model, the variance of a trait can be decomposed into genetic and residual components.

Table 7: Full twin and SNP heritability results:

Measure	Twin h2	SNP h2	Twin SE	Twin 95% CI	SNP SE	SNP 95% CI	Twin N (pairs)	SNP N (indiv)	p (SNP h2)
**Stable CFA score (simultaneous)**	0.701	n/a	0.047	[0.608-0.792]	n/a	n/a	6110	n/a	n/a
**Stable CFA score (extracted FS)**	0.760	0.14	0.023	[0.715-0.805]	0.049	[0.044-0.236]	6110	6002	0.002*
**Stable IRT score (simultaneous)**	0.611	n/a	0.043	[0.527-0.695]	n/a	n/a	6110	n/a	n/a
**Stable IRT score (extracted FS)**	0.620	0.095	0.023	[0.575-0.665]	0.048	[0.001-0.189]	6110	6001	0.025*
Parent-rated SDQ anxiety at 7	0.475	0.009	0.037	[0.402-0.548]	0.053	[-0.095-0.113]	5359	5272	0.438
Teacher-rated SDQ anxiety at 7	0.509	0.126	0.018	[0.474-0.544]	0.065	[-0.001-0.253]	4419	4336	0.023*
Self-rated MFQ at 12	0.283	0.039	0.051	[0.183-0.383]	0.064	[-0.086-0.164]	4490	4414	0.27
Self-rated SDQ anxiety at 12	0.399	0.082	0.026	[0.348-0.450]	0.065	[-0.045-0.209]	4475	4396	0.106
Parent-rated SDQ anxiety at 12	0.531	0.022	0.041	[0.451-0.611]	0.062	[-0.100-0.144]	4482	4408	0.364
Parent-rated MFQ at 12	0.478	0.029	0.038	[0.404-0.552]	0.063	[-0.094-0.152]	4482	4407	0.326
Teacher-rated SDQ anxiety at 12	0.426	0.029	0.023	[0.381-0.471]	0.077	[-0.122-0.180]	3695	3652	0.359
Self-rated SDQ anxiety at 16	0.428	0.094	0.018	[0.393-0.463]	0.075	[-0.053-0.241]	3822	3759	0.108
Self-rated CASI at 16	0.445	0.069	0.019	[0.408-0.482]	0.076	[-0.080-0.218]	3824	3760	0.187
Self-rated MFQ at 16	0.403	0	0.024	[0.356-0.450]	0.074	[-0.145-0.145]	3824	3761	0.5
Parent-rated MFQ at 16	0.567	0.099	0.031	[0.506-0.628]	0.073	[-0.044-0.242]	3838	3774	0.08
Parent-rated ARBQ at age 16	0.413	0	0.039	[0.337-0.489]	0.066	[-0.129-0.129]	3840	3775	0.499

Note: ‘h2’ = heritability; ’SE’ = standard error; ‘95% CI’ = 95% confidence intervals; ‘p’ = the p-value associated with the SNP heritability estimate, and whether $p<0.05$ (indicated with ‘*’); ‘FS’ = stable emotional problems factor scores extracted from latent modelling; ‘simultaneous’ = simultaneous latent trait-twin modelling.

Figure 4: Twin and SNP heritability results for a simple mean approach to aggregating emotional problems measures, with sensitivity analyses to investigate rater effects

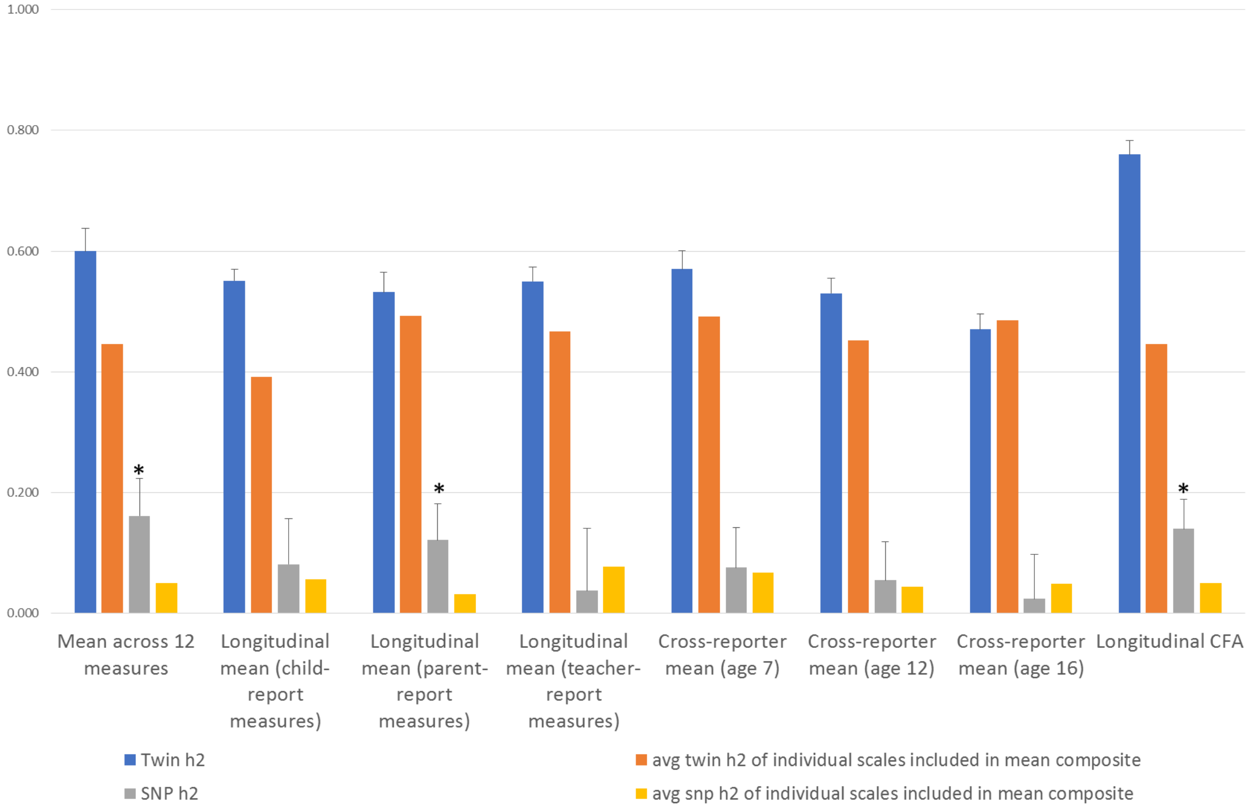

Note: means were calculated only for individuals with data present on at least half of the variables. Twin and SNP heritability results for the means (blue and grey) are compared to average heritabilities of the constituent individual measures (orange and yellow). '*' indicates a significant SNP heritability estimate.

Supplementary Figure 4 shows that the simpler approach of creating a mean of the 12 anxiety and depression measures yields similar results to a latent modelling approach (comparing the first and eighth sections of the plot). As such, the finding of higher twin and SNP heritabilities holds for even a crude composite measure that adds up common *and* measure-specific error (rather than latent modelling to remove error). This bolsters the argument that aggregating across time and across raters taps into a more heritable, core 'trait' aspect of emotional development.

To explore the whether the effect of extracting longitudinal stability increasing heritability remained when using single-rater composites, we created longitudinal means for child-, parent- and teacher-reports separately (sections 2-4 of the plot). Results suggest that this is the case. Twin estimates increased from ~45% to ~55%. SNP heritability point estimates increased for child- and parent-report longitudinal means (with longitudinal aggregation boosting SNP heritability of parent-report measures into statistical significance), but not for the teacher-report composite. Given the similar point estimates and large standard error intervals around SNP heritability estimates, it would be inappropriate to compare SNP heritability estimates.

To investigate the contribution of trans-situational variance (i.e. rater agreement on childhood emotional problems), we created means of anxiety and depression measures across reporters but at individual ages (sections 5-7 of the plot). Results suggest that combining cross-sectional measures across reporters increases twin and SNP heritability estimates at ages 7 and 12, but

not at 16. Again, standard errors for SNP heritability estimates are large, such that we cannot compare estimates for different composite measures, and we cannot say that aggregation across raters *significantly* increases SNP heritability. The heritability increase resulting from aggregating measures using a simple mean approach is weaker for SNP heritability than for twin estimates, but this is partly because the sample sizes for SNP heritability estimation were ~4000 (rather than over 6000 for latent modelling with maximum likelihood). The restriction of the mean composite approach to individuals with complete data is a disadvantage in comparison to the latent modelling approach. The finding that latent modelling is more powerful than simple approaches replicates evidence from the Netherlands Twin Register: a latent anxious depression phenotype was substantially more heritable than the scores observed at any age, but measure created by simply summing data from the different ages was considerably less heritable (Lubke et al. 2016).

Overall, results suggest that both the mean composite and latent factor across time and across reporters shows increased heritability as a result of tapping into longitudinally stable aspects of behaviour that are also agreed upon by different raters. Future research could more thoroughly and explicitly test the structure of age-, scale-, and rater-specific influences on emotional problems. For example, in the Trait-State-Occasion model, significant covariances between observed variable error terms indicates that trait stability is inflated by shared method variance cross waves.

Investigation of the contribution of individuals with persistent emotional problems to the increased heritability of our stable phenotype.

In studies of adults, recurrent depression is more severe and heritable than single episode depression. It follows that the heritability for stable childhood emotional problems could be higher because we are capturing the higher heritability of severe recurrent symptoms, rather than stability at any level of emotional symptoms. This is especially worth investigating given the high genetic correlation of our stable childhood emotional problems phenotype with adult depression.

To test this idea, we calculated how many of the 12 measures each individual was an outlier for. Outliers were defined as individuals with z-scores >=1.96 (i.e. cutting off the top 1% of scores). We removed twin pairs for whom twin 1 or 2 was an outlier for >6 of the 12 scales (i.e. removed 21 pairs). Then, we re-calculated the twin heritability of a single common factor (from CFA).

We found a lower twin heritability for stable emotional problems when excluding individuals with persistent problems (0.50 (se=0.05) vs 0.70 (se=0.05) for the CFA factor scores using all 6110 pairs). This suggests that a small number of young people with enduring severe emotional symptoms could be disproportionately contributing to the high heritability of stable emotional problems. However, 95% CIs for the two estimates just overlap (.40-.60 and .60-.80), so the difference is not statistically significant.

Adjusting the definition of outliers to z>=1.64 (the top 5% of scores) led to the removal of 43 twin pairs for whom at least one twin was an outlier on more than half of the measures. The heritability estimate hardly decreased below 0.50 (0.49 (se=0.05)), but the upper 95% CI decreased to .57, such that the CIs no longer overlapped with the estimates from the full sample. Overall, there is no strong evidence that individuals with severe persistent symptoms are inflating heritability estimates.

Figures 5 and 6: Polygenic score results

The PRSice 2 plots below demonstrate that the R^2 is not dramatically better at the best p-value threshold than at the others. This is indicative of reliable prediction for a polygenic trait.

<u>Figure 4: Prediction of CFA scores using UK Biobank anxiety polygenic score</u>

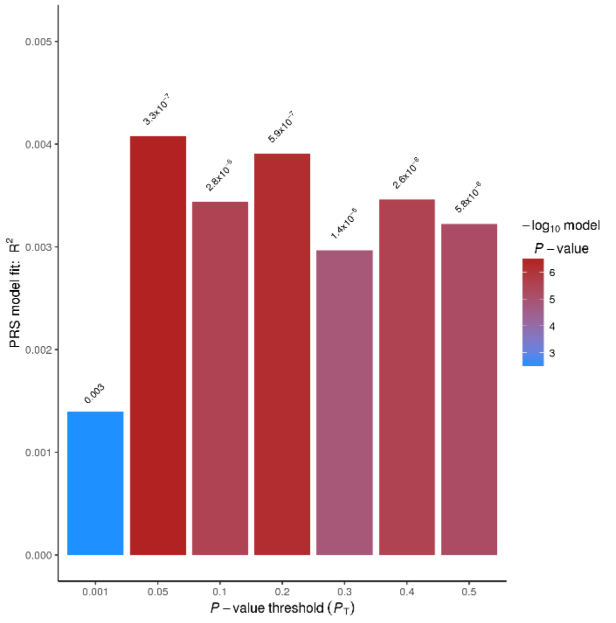

<u>Figure 5: Prediction of IRT scores using UK Biobank anxiety polygenic score</u>

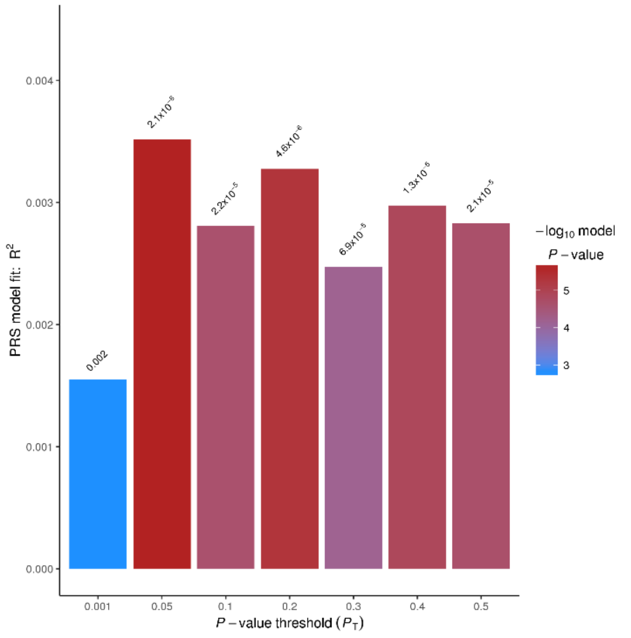

Table 8: variance explained by polygenic scores for MDD, derived from summary statistics for a subset of 10,000 variants in the PGC sample including 23&Me.

	R^2	P-value	Empirical P-value
Stable CFA score	0.0041	0.0000	0.0001*
Stable IRT score	0.0031	0.0000	0.0002*
Parent-rated SDQ at 7	0.0006	0.0754	0.1268
Teacher-rated SDQ at 7	0.0023	0.0015	0.0026*
Self-rated MFQ at 12	0.0051	0.0000	0.0001*
Self-rated SDQ at 12	0.0024	0.0010	0.0020*
Parent-rated SDQ at 12	0.0009	0.0411	0.0743
Parent-rated MFQ at 12	0.0014	0.0113	0.0213*
Teacher-rated SDQ at 12	0.0005	0.1633	0.2582
Self-rated SDQ at 16	0.0022	0.0021	0.0038*
Self-rated CASI at 16	0.0034	0.0001	0.0005*
Self-rated MFQ at 16	0.0020	0.0042	0.0091*
Parent-rated MFQ at 16	0.0014	0.0202	0.0366*
Parent-rated ARBQ at 16	0.0021	0.0036	0.0067*

Note: '*' indicates target TEDS phenotypes that were significantly predicted by polygenic scores at the more stringent empirical p-value thresholds.

Figures 7 and 8: Genome-wide association results

No significant SNPs were identified. There are no SNPs that exceed a –log10 p-value of more than 6. This was expected, given the relatively small sample (6110), and the lack of associations in previous, similarly powered studies of emotional problems (e.g. Benke et al., 2014 meta-analysis of preschool emotional problems, N=4596).

IRT score GWAS: FUMA plot:

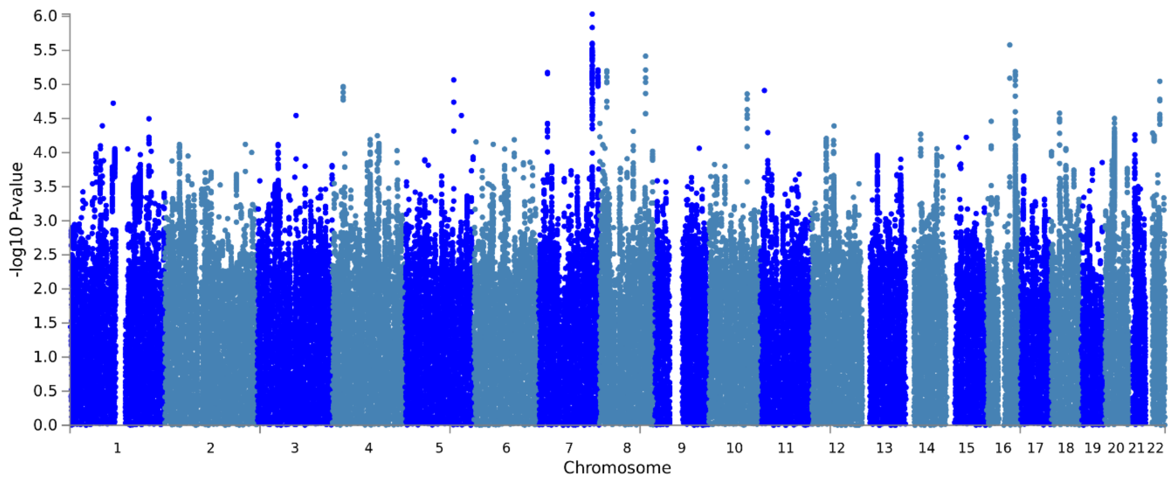

CFA score GWAS: FUMA plot:

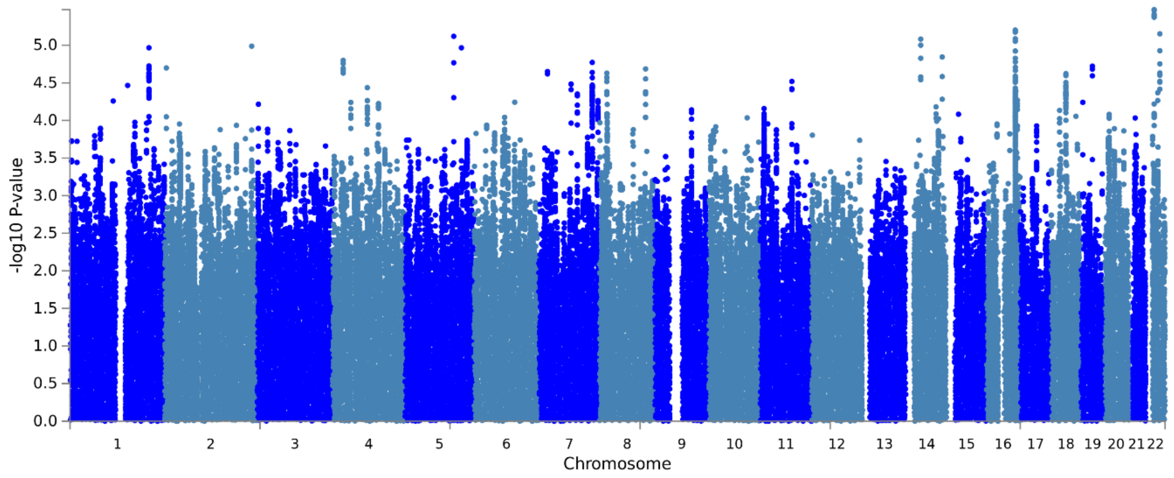

Table 9: Genetic correlations between stable emotional problems (from CFA) and psychiatric traits, obtained using LD Score regression.

**Trait**	**PMID**	**rg**	**se**	**z**	**p**	**h2_obs**	**h2_obs_se**
Depressive symptoms	27089181	0.64	0.19	3.37	0.0008**	0.05	0
Adult depression (PGC) †		0.48	0.14	3.35	0.0008**	0	0
Adult anxiety (UK Biobank) †		0.45	0.15	2.92	0.0035**	0.11	0.01
Subjective well being	27089181	-0.36	0.15	-2.36	0.0182*	0.02	0
Major depressive disorder	22472876	0.37	0.2	1.9	0.0574	0.18	0.03
Schizophrenia	25056061	0.16	0.09	1.8	0.0718	0.45	0.02
PGC cross-disorder analysis	23453885	0.21	0.14	1.49	0.1368	0.18	0.01
Attention deficit hyperactivity disorder (GC)	27663945	0.34	0.3	1.14	0.2528	0.08	0.03
Attention deficit hyperactivity disorder (No GC)	27663945	0.34	0.3	1.14	0.2538	0.08	0.03
Attention deficit hyperactivity disorder	20732625	-0.27	0.3	-0.9	0.3677	0.22	0.1
Autism spectrum disorder	0	-0.06	0.15	-0.39	0.6972	0.46	0.05
Bipolar disorder	21926972	0.02	0.14	0.14	0.8857	0.45	0.04
Anorexia Nervosa	24514567	-0.01	0.13	-0.06	0.9528	0.4	0.04

Note: Pairs of traits are ordered according to the p-value of their genetic correlation. * = significant at p<0.05, **= significant at p<0.00385 i.e. (0.05/13) to correct for multiple tests; †= genetic correlation analysis performed separately (using LDSR) because phenotype was *not* in LD Hub.

The genetic correlations between our CFA-derived stable scores, and case-control depression, depressive symptoms, case-control generalised anxiety, and wellbeing, are significant at p<0.05. The negative genetic correlation with wellbeing did not remain significant at the more stringent threshold (p<0. 00385). Genetic correlations have many possible interpretations other than true biological pleiotropy (Martin et al. 2017). For example, the relationship between adult depression and childhood stable emotional problems could be mediated by shared genetic risk with low levels of well-being. It has been suggested that the genetic correlation between MDD and depressive symptoms in the population could be accounted for by shared genetic risk with low levels of subjective well-being (Direk et al. 2017). Another issue is that nosological issues involving heterogeneity, comorbidity and misclassification might inflate phenotypic and genetic correlations.

Full authors list: Major Depressive Disorder Working Group of the Psychiatric Genomics Consortium

Naomi R Wray* 1, 2
Stephan Ripke* 3, 4, 5
Manuel Mattheisen* 6, 7, 8, 9
Maciej Trzaskowski* 1
Enda M Byrne 1
Abdel Abdellaoui 10
Mark J Adams 11
Esben Agerbo 9, 12, 13
Tracy M Air 14
Till F M Andlauer 15, 16
Silviu-Alin Bacanu 17
Marie Bækvad-Hansen 9, 18
Aartjan T F Beekman 19
Tim B Bigdeli 17, 20
Elisabeth B Binder 15, 21
Douglas H R Blackwood 11
Julien Bryois 22
Henriette N Buttenschøn 8, 9, 23
Jonas Bybjerg-Grauholm 9, 18
Na Cai 24, 25
Enrique Castelao 26
Jane Hvarregaard Christensen 7, 8, 9
Toni-Kim Clarke 11
Jonathan R I Coleman 27
Lucía Colodro-Conde 28
Baptiste Couvy-Duchesne 29, 30
Nick Craddock 31
Gregory E Crawford 32, 33
Gail Davies 34
Ian J Deary 34
Franziska Degenhardt 35, 36
Eske M Derks 28
Nese Direk 37, 38
Conor V Dolan 10
Erin C Dunn 39, 40, 41
Thalia C Eley 27
Valentina Escott-Price 42
Farnush Farhadi Hassan Kiadeh 43
Hilary K Finucane 44, 45
Andreas J Forstner 35, 36, 46, 47
Josef Frank 48
Héléna A Gaspar 27
Michael Gill 49
Fernando S Goes 50
Scott D Gordon 51
Jakob Grove 7, 8, 9, 52
Lynsey S Hall 11, 53
Christine Søholm Hansen 9, 18
Thomas F Hansen 54, 55, 56
Stefan Herms 35, 36, 47
Ian B Hickie 57
Per Hoffmann 35, 36, 47
Georg Homuth 58
Carsten Horn 59
Jouke-Jan Hottenga 10
David M Hougaard 9, 18
Marcus Ising 60
Rick Jansen 19, 19
Eric Jorgenson 61
James A Knowles 62
Isaac S Kohane 63, 64, 65
Julia Kraft 4
Warren W. Kretzschmar 66
Jesper Krogh 67
Zoltán Kutalik 68, 69
Yihan Li 66
Penelope A Lind 28
Donald J MacIntyre 70, 71
Dean F MacKinnon 50
Robert M Maier 2
Wolfgang Maier 72
Jonathan Marchini 73
Hamdi Mbarek 10
Patrick McGrath 74
Peter McGuffin 27
Sarah E Medland 28
Divya Mehta 2, 75
Christel M Middeldorp 10, 76, 77
Evelin Mihailov 78
Yuri Milaneschi 19, 19
Lili Milani 78
Francis M Mondimore 50
Grant W Montgomery 1
Sara Mostafavi 79, 80
Niamh Mullins 27
Matthias Nauck 81, 82
Bernard Ng 80
Michel G Nivard 10
Dale R Nyholt 83
Paul F O'Reilly 27
Hogni Oskarsson 84
Michael J Owen 85
Jodie N Painter 28
Carsten Bøcker Pedersen 9, 12, 13
Marianne Giørtz Pedersen 9, 12, 13
Roseann E. Peterson 17, 86
Erik Pettersson 22
Wouter J Peyrot 19
Giorgio Pistis 26
Danielle Posthuma 87, 88
Jorge A Quiroz 89
Per Qvist 7, 8, 9
John P Rice 90
Brien P. Riley 17
Margarita Rivera 27, 91
Saira Saeed Mirza 37
Robert Schoevers 92
Eva C Schulte 93, 94
Ling Shen 61
Jianxin Shi 95
Stanley I Shyn 96
Engilbert Sigurdsson 97
Grant C B Sinnamon 98
Johannes H Smit 19
Daniel J Smith 99
Hreinn Stefansson 100
Stacy Steinberg 100
Fabian Streit 48
Jana Strohmaier 48
Katherine E Tansey 101
Henning Teismann 102
Alexander Teumer 103
Wesley Thompson 9, 55, 104, 105
Pippa A Thomson 106
Thorgeir E Thorgeirsson 100
Matthew Traylor 107
Jens Treutlein 48
Vassily Trubetskoy 4

André G Uitterlinden 108
Daniel Umbricht 109
Sandra Van der Auwera 110
Albert M van Hemert 111
Alexander Viktorin 22
Peter M Visscher 1, 2
Yunpeng Wang 9, 55, 105
Bradley T. Webb 112
Shantel Marie Weinsheimer 9, 55
Jürgen Wellmann 102
Gonneke Willemsen 10
Stephanie H Witt 48
Yang Wu 1
Hualin S Xi 113
Jian Yang 2, 114
Futao Zhang 1
Volker Arolt 115
Bernhard T Baune 14
Klaus Berger 102
Dorret I Boomsma 10
Sven Cichon 35, 47, 116, 117
Udo Dannlowski 115
EJC de Geus 10, 118
J Raymond DePaulo 50
Enrico Domenici 119
Katharina Domschke 120
Tõnu Esko 5, 78
Hans J Grabe 110
Steven P Hamilton 121
Caroline Hayward 122
Andrew C Heath 90
Kenneth S Kendler 17
Stefan Kloiber 60, 123, 124
Glyn Lewis 125
Qingqin S Li 126
Susanne Lucae 60
Pamela AF Madden 90
Patrik K Magnusson 22
Nicholas G Martin 51
Andrew M McIntosh 11, 34
Andres Metspalu 78, 127
Ole Mors 9, 128
Preben Bo Mortensen 8, 9, 12, 13
Bertram Müller-Myhsok 15, 16, 129
Merete Nordentoft 9, 130
Markus M Nöthen 35, 36
Michael C O'Donovan 85
Sara A Paciga 131
Nancy L Pedersen 22
Brenda WJH Penninx 19
Roy H Perlis 39, 132
David J Porteous 106
James B Potash 133
Martin Preisig 26
Marcella Rietschel 48
Catherine Schaefer 61
Thomas G Schulze 48, 94, 134, 135, 136
Jordan W Smoller 39, 40, 41
Kari Stefansson 100, 137
Henning Tiemeier 37, 138, 139
Rudolf Uher 140
Henry Völzke 103
Myrna M Weissman 74, 141
Thomas Werge 9, 55, 142
Cathryn M Lewis* 27, 143
Douglas F Levinson* 144
Gerome Breen* 27, 145
Anders D Børglum* 7, 8, 9
Patrick F Sullivan* 22, 146, 147,

1, Institute for Molecular Bioscience, The University of Queensland, Brisbane, QLD, AU
2, Queensland Brain Institute, The University of Queensland, Brisbane, QLD, AU
3, Analytic and Translational Genetics Unit, Massachusetts General Hospital, Boston, MA, US
4, Department of Psychiatry and Psychotherapy, Universitätsmedizin Berlin Campus Charité Mitte, Berlin, DE
5, Medical and Population Genetics, Broad Institute, Cambridge, MA, US
6, Centre for Psychiatry Research, Department of Clinical Neuroscience, Karolinska Institutet, Stockholm, SE
7, Department of Biomedicine, Aarhus University, Aarhus, DK
8, iSEQ, Centre for Integrative Sequencing, Aarhus University, Aarhus, DK
9, iPSYCH, The Lundbeck Foundation Initiative for Integrative Psychiatric Research, DK
10, Dept of Biological Psychology & EMGO+ Institute for Health and Care Research, Vrije Universiteit Amsterdam, Amsterdam, NL
11, Division of Psychiatry, University of Edinburgh, Edinburgh, GB
12, Centre for Integrated Register-based Research, Aarhus University, Aarhus, DK
13, National Centre for Register-Based Research, Aarhus University, Aarhus, DK
14, Discipline of Psychiatry, University of Adelaide, Adelaide, SA, AU
15, Department of Translational Research in Psychiatry, Max Planck Institute of Psychiatry, Munich, DE
16, Munich Cluster for Systems Neurology (SyNergy), Munich, DE
17, Department of Psychiatry, Virginia Commonwealth University, Richmond, VA, US
18, Center for Neonatal Screening, Department for Congenital Disorders, Statens Serum Institut, Copenhagen, DK
19, Department of Psychiatry, Vrije Universiteit Medical Center and GGZ inGeest, Amsterdam, NL
20, Virginia Institute for Psychiatric and Behavior Genetics, Richmond, VA, US
21, Department of Psychiatry and Behavioral Sciences, Emory University School of Medicine, Atlanta, GA, US
22, Department of Medical Epidemiology and Biostatistics, Karolinska Institutet, Stockholm, SE
23, Department of Clinical Medicine, Translational Neuropsychiatry Unit, Aarhus University, Aarhus, DK
24, Human Genetics, Wellcome Trust Sanger Institute, Cambridge, GB
25, Statistical genomics and systems genetics, European Bioinformatics Institute (EMBL-EBI), Cambridge, GB
26, Department of Psychiatry, University Hospital of Lausanne, Prilly, Vaud, CH
27, MRC Social Genetic and Developmental Psychiatry Centre, King's College London, London, GB
28, Genetics and Computational Biology, QIMR Berghofer Medical Research Institute, Herston, QLD, AU
29, Centre for Advanced Imaging, The University of Queensland, Saint Lucia, QLD, AU
30, Queensland Brain Institute, The University of Queensland, Saint Lucia, QLD, AU
31, Psychological Medicine, Cardiff University, Cardiff, GB
32, Center for Genomic and Computational Biology, Duke University, Durham, NC, US
33, Department of Pediatrics, Division of Medical Genetics, Duke University, Durham, NC, US
34, Centre for Cognitive Ageing and Cognitive Epidemiology, University of Edinburgh, Edinburgh, GB
35, Institute of Human Genetics, University of Bonn, Bonn, DE
36, Life&Brain Center, Department of Genomics, University of Bonn, Bonn, DE
37, Epidemiology, Erasmus MC, Rotterdam, Zuid-Holland, NL
38, Psychiatry, Dokuz Eylul University School Of Medicine, Izmir, TR

39, Department of Psychiatry, Massachusetts General Hospital, Boston, MA, US
40, Psychiatric and Neurodevelopmental Genetics Unit (PNGU), Massachusetts General Hospital, Boston, MA, US
41, Stanley Center for Psychiatric Research, Broad Institute, Cambridge, MA, US
42, Neuroscience and Mental Health, Cardiff University, Cardiff, GB
43, Bioinformatics, University of British Columbia, Vancouver, BC, CA
44, Department of Epidemiology, Harvard T.H. Chan School of Public Health, Boston, MA, US
45, Department of Mathematics, Massachusetts Institute of Technology, Cambridge, MA, US
46, Department of Psychiatry (UPK), University of Basel, Basel, CH
47, Human Genomics Research Group, Department of Biomedicine, University of Basel, Basel, CH
48, Department of Genetic Epidemiology in Psychiatry, Central Institute of Mental Health, Medical Faculty Mannheim, Heidelberg University, Mannheim, Baden-Württemberg, DE
49, Department of Psychiatry, Trinity College Dublin, Dublin, IE
50, Psychiatry & Behavioral Sciences, Johns Hopkins University, Baltimore, MD, US
51, Genetics and Computational Biology, QIMR Berghofer Medical Research Institute, Brisbane, QLD, AU
52, Bioinformatics Research Centre, Aarhus University, Aarhus, DK
53, Institute of Genetic Medicine, Newcastle University, Newcastle upon Tyne, GB
54, Danish Headache Centre, Department of Neurology, Rigshospitalet, Glostrup, DK
55, Institute of Biological Psychiatry, Mental Health Center Sct. Hans, Mental Health Services Capital Region of Denmark, Copenhagen, DK
56, iPSYCH, The Lundbeck Foundation Initiative for Psychiatric Research, Copenhagen, DK
57, Brain and Mind Centre, University of Sydney, Sydney, NSW, AU
58, Interfaculty Institute for Genetics and Functional Genomics, Department of Functional Genomics, University Medicine and Ernst Moritz Arndt University Greifswald, Greifswald, Mecklenburg-Vorpommern, DE
59, Roche Pharmaceutical Research and Early Development, Pharmaceutical Sciences, Roche Innovation Center Basel, F. Hoffmann-La Roche Ltd, Basel, CH
60, Max Planck Institute of Psychiatry, Munich, DE
61, Division of Research, Kaiser Permanente Northern California, Oakland, CA, US
62, Psychiatry & The Behavioral Sciences, University of Southern California, Los Angeles, CA, US
63, Department of Biomedical Informatics, Harvard Medical School, Boston, MA, US
64, Department of Medicine, Brigham and Women's Hospital, Boston, MA, US
65, Informatics Program, Boston Children's Hospital, Boston, MA, US
66, Wellcome Trust Centre for Human Genetics, University of Oxford, Oxford, GB
67, Department of Endocrinology at Herlev University Hospital, University of Copenhagen, Copenhagen, DK
68, Institute of Social and Preventive Medicine (IUMSP), University Hospital of Lausanne, Lausanne, VD, CH
69, Swiss Institute of Bioinformatics, Lausanne, VD, CH
70, Division of Psychiatry, Centre for Clinical Brain Sciences, University of Edinburgh, Edinburgh, GB
71, Mental Health, NHS 24, Glasgow, GB
72, Department of Psychiatry and Psychotherapy, University of Bonn, Bonn, DE
73, Statistics, University of Oxford, Oxford, GB
74, Psychiatry, Columbia University College of Physicians and Surgeons, New York, NY, US
75, School of Psychology and Counseling, Queensland University of Technology, Brisbane, QLD, AU

76, Child and Youth Mental Health Service, Children's Health Queensland Hospital and Health Service, South Brisbane, QLD, AU
77, Child Health Research Centre, University of Queensland, Brisbane, QLD, AU
78, Estonian Genome Center, University of Tartu, Tartu, EE
79, Medical Genetics, University of British Columbia, Vancouver, BC, CA
80, Statistics, University of British Columbia, Vancouver, BC, CA
81, DZHK (German Centre for Cardiovascular Research), Partner Site Greifswald, University Medicine, University Medicine Greifswald, Greifswald, Mecklenburg-Vorpommern, DE
82, Institute of Clinical Chemistry and Laboratory Medicine, University Medicine Greifswald, Greifswald, Mecklenburg-Vorpommern, DE
83, Institute of Health and Biomedical Innovation, Queensland University of Technology, Brisbane, QLD, AU
84, Humus, Reykjavik, IS
85, MRC Centre for Neuropsychiatric Genetics and Genomics, Cardiff University, Cardiff, GB
86, Virginia Institute for Psychiatric & Behavioral Genetics, Virginia Commonwealth University, Richmond, VA, US
87, Clinical Genetics, Vrije Universiteit Medical Center, Amsterdam, NL
88, Complex Trait Genetics, Vrije Universiteit Amsterdam, Amsterdam, NL
89, Solid Biosciences, Boston, MA, US
90, Department of Psychiatry, Washington University in Saint Louis School of Medicine, Saint Louis, MO, US
91, Department of Biochemistry and Molecular Biology II, Institute of Neurosciences, Center for Biomedical Research, University of Granada, Granada, ES
92, Department of Psychiatry, University of Groningen, University Medical Center Groningen, Groningen, NL
93, Department of Psychiatry and Psychotherapy, Medical Center of the University of Munich, Campus Innenstadt, Munich, DE
94, Institute of Psychiatric Phenomics and Genomics (IPPG), Medical Center of the University of Munich, Campus Innenstadt, Munich, DE
95, Division of Cancer Epidemiology and Genetics, National Cancer Institute, Bethesda, MD, US
96, Behavioral Health Services, Kaiser Permanente Washington, Seattle, WA, US
97, Faculty of Medicine, Department of Psychiatry, University of Iceland, Reykjavik, IS
98, School of Medicine and Dentistry, James Cook University, Townsville, QLD, AU
99, Institute of Health and Wellbeing, University of Glasgow, Glasgow, GB
100, deCODE Genetics / Amgen, Reykjavik, IS
101, College of Biomedical and Life Sciences, Cardiff University, Cardiff, GB
102, Institute of Epidemiology and Social Medicine, University of Münster, Münster, Nordrhein-Westfalen, DE
103, Institute for Community Medicine, University Medicine Greifswald, Greifswald, Mecklenburg-Vorpommern, DE
104, Department of Psychiatry, University of California, San Diego, San Diego, CA, US
105, KG Jebsen Centre for Psychosis Research, Norway Division of Mental Health and Addiction, Oslo University Hospital, Oslo, NO
106, Medical Genetics Section, CGEM, IGMM, University of Edinburgh, Edinburgh, GB
107, Clinical Neurosciences, University of Cambridge, Cambridge, GB
108, Internal Medicine, Erasmus MC, Rotterdam, Zuid-Holland, NL
109, Roche Pharmaceutical Research and Early Development, Neuroscience, Ophthalmology and Rare Diseases Discovery & Translational Medicine Area, Roche Innovation Center Basel, F. Hoffmann-La Roche Ltd, Basel, CH

110, Department of Psychiatry and Psychotherapy, University Medicine Greifswald, Greifswald, Mecklenburg-Vorpommern, DE
111, Department of Psychiatry, Leiden University Medical Center, Leiden, NL
112, Virginia Institute of Psychiatric & Behavioral Genetics, Virginia Commonwealth University, Richmond, VA, US
113, Computational Sciences Center of Emphasis, Pfizer Global Research and Development, Cambridge, MA, US
114, Institute for Molecular Bioscience; Queensland Brain Institute, The University of Queensland, Brisbane, QLD, AU
115, Department of Psychiatry, University of Münster, Münster, Nordrhein-Westfalen, DE
116, Institute of Medical Genetics and Pathology, University Hospital Basel, University of Basel, Basel, CH
117, Institute of Neuroscience and Medicine (INM-1), Research Center Juelich, Juelich, DE
118, Amsterdam Public Health Institute, Vrije Universiteit Medical Center, Amsterdam, NL
119, Centre for Integrative Biology, Università degli Studi di Trento, Trento, Trentino-Alto Adige, IT
120, Department of Psychiatry and Psychotherapy, Medical Center, University of Freiburg, Faculty of Medicine, University of Freiburg, Freiburg, DE
121, Psychiatry, Kaiser Permanente Northern California, San Francisco, CA, US
122, Medical Research Council Human Genetics Unit, Institute of Genetics and Molecular Medicine, University of Edinburgh, Edinburgh, GB
123, Department of Psychiatry, University of Toronto, Toronto, ON, CA
124, Centre for Addiction and Mental Health, Toronto, ON, CA
125, Division of Psychiatry, University College London, London, GB
126, Neuroscience Therapeutic Area, Janssen Research and Development, LLC, Titusville, NJ, US
127, Institute of Molecular and Cell Biology, University of Tartu, Tartu, EE
128, Psychosis Research Unit, Aarhus University Hospital, Risskov, Aarhus, DK
129, University of Liverpool, Liverpool, GB
130, Mental Health Center Copenhagen, Copenhagen University Hospital, Copenhagen, DK
131, Human Genetics and Computational Biomedicine, Pfizer Global Research and Development, Groton, CT, US
132, Psychiatry, Harvard Medical School, Boston, MA, US
133, Psychiatry, University of Iowa, Iowa City, IA, US
134, Department of Psychiatry and Behavioral Sciences, Johns Hopkins University, Baltimore, MD, US
135, Department of Psychiatry and Psychotherapy, University Medical Center Göttingen, Goettingen, Niedersachsen, DE
136, Human Genetics Branch, NIMH Division of Intramural Research Programs, Bethesda, MD, US
137, Faculty of Medicine, University of Iceland, Reykjavik, IS
138, Child and Adolescent Psychiatry, Erasmus MC, Rotterdam, Zuid-Holland, NL
139, Psychiatry, Erasmus MC, Rotterdam, Zuid-Holland, NL
140, Psychiatry, Dalhousie University, Halifax, NS, CA
141, Division of Epidemiology, New York State Psychiatric Institute, New York, NY, US
142, Department of Clinical Medicine, University of Copenhagen, Copenhagen, DK
143, Department of Medical & Molecular Genetics, King's College London, London, GB
144, Psychiatry & Behavioral Sciences, Stanford University, Stanford, CA, US
145, NIHR BRC for Mental Health, King's College London, London, GB
146, Genetics, University of North Carolina at Chapel Hill, Chapel Hill, NC, US
147, Psychiatry, University of North Carolina at Chapel Hill, Chapel Hill, NC, US

LD Hub acknowledgements

We gratefully acknowledge all the studies and databases that made GWAS summary data available: **ADIPOGen** (Adiponectin genetics consortium), **C4D** (Coronary Artery Disease Genetics Consortium), **CARDIoGRAM** (Coronary ARtery DIsease Genome wide Replication and Meta-analysis), **CKDGen** (Chronic Kidney Disease Genetics consortium), **dbGAP** (database of Genotypes and Phenotypes), **DIAGRAM** (DIAbetes Genetics Replication And Meta-analysis), **ENIGMA** (Enhancing Neuro Imaging Genetics through Meta Analysis), **EAGLE** (EArly Genetics & Lifecourse Epidemiology Eczema Consortium, excluding 23andMe), **EGG** (Early Growth Genetics Consortium), **GABRIEL** (A Multidisciplinary Study to Identify the Genetic and Environmental Causes of Asthma in the European Community), **GCAN** (Genetic Consortium for Anorexia Nervosa), **GEFOS** (GEnetic Factors for OSteoporosis Consortium), **GIANT** (Genetic Investigation of ANthropometric Traits), **GIS** (Genetics of Iron Status consortium

), **GLGC** (Global Lipids Genetics Consortium), **GPC** (Genetics of Personality Consortium), **GUGC** (Global Urate and Gout consortium), **HaemGen** (haemotological and platelet traits genetics consortium), **HRgene** (Heart Rate consortium), **IIBDGC** (International Inflammatory Bowel Disease Genetics Consortium), **ILCCO** (International Lung Cancer Consortium), **IMSGC** (International Multiple Sclerosis Genetic Consortium), **MAGIC** (Meta-Analyses of Glucose and Insulin-related traits Consortium), **MESA** (Multi-Ethnic Study of Atherosclerosis), **PGC** (Psychiatric Genomics Consortium), **Project MinE** consortium, **ReproGen** (Reproductive Genetics Consortium), **SSGAC (**Social Science Genetics Association Consortium) and **TAG** (Tobacco and Genetics Consortium), **TRICL** (Transdisciplinary Research in Cancer of the Lung consortium), **UK Biobank**.

We gratefully acknowledge the contributions of Alkes Price (the systemic lupus erythematosus GWAS and primary biliary cirrhosis GWAS) and Johannes Kettunen (lipids metabolites GWAS).

Bibliography

Chang, C.C., Chow, C.C., Tellier, L.C., Vattikuti, S., Purcell, S.M. and Lee, J.J. 2015. Second-generation PLINK: rising to the challenge of larger and richer datasets. *GigaScience* 4, p. 7.

Danecek, P., Auton, A., Abecasis, G., Albers, C.A., Banks, E., DePristo, M.A., Handsaker, R.E., Lunter, G., Marth, G.T., Sherry, S.T., McVean, G., Durbin, R. and 1000 Genomes Project Analysis Group 2011. The variant call format and VCFtools. *Bioinformatics* 27(15), pp. 2156–2158.

Direk, N., Williams, S., Smith, J.A., Ripke, S., Sullivan, P.F., et al. 2017. An Analysis of Two Genome-wide Association Meta-analyses Identifies a New Locus for Broad Depression Phenotype. *Biological Psychiatry* 82(5), pp. 322–329.

Fuchsberger, C., Abecasis, G.R. and Hinds, D.A. 2015. minimac2: faster genotype imputation. *Bioinformatics* 31(5), pp. 782–784.

GBIF.ORG R: a language and environment for statistical computing [Online]. Available at: http://www.gbif.org/resource/81287 [Accessed: 8 May 2017].

Howie, B., Fuchsberger, C., Stephens, M., Marchini, J. and Abecasis, G.R. 2012. Fast and accurate genotype imputation in genome-wide association studies through pre-phasing. *Nature Genetics* 44(8), pp. 955–959.

International HapMap 3 Consortium, Altshuler, D.M., Gibbs, R.A., Peltonen, L., McEwen, J.E., et al. 2010. Integrating common and rare genetic variation in diverse human populations. *Nature* 467(7311), pp. 52–58.

Lubke, G.H., Miller, P.J., Verhulst, B., Bartels, M., van Beijsterveldt, T., Willemsen, G., Boomsma, D.I. and Middeldorp, C.M. 2016. A powerful phenotype for gene-finding studies derived from trajectory analyses of symptoms of anxiety and depression between age seven and 18. *American Journal of Medical Genetics. Part B, Neuropsychiatric Genetics* 171(7), pp. 948–957.

Martin, J., Taylor, M.J. and Lichtenstein, P. 2017. Assessing the evidence for shared genetic risks across psychiatric disorders and traits. *Psychological Medicine*, pp. 1–16.

Purcell, S., Neale, B., Todd-Brown, K., Thomas, L., Ferreira, M.A.R., Bender, D., Maller, J., Sklar, P., de Bakker, P.I.W., Daly, M.J. and Sham, P.C. 2007. PLINK: a tool set for whole-genome association and population-based linkage analyses. *American Journal of Human Genetics* 81(3), pp. 559–575.
